# Recent Advances in Electrodeposition of Nickel-Based Nanocomposites Enhanced with Lubricating Nanoparticles

**DOI:** 10.1007/s41871-024-00245-6

**Published:** 2024-12-12

**Authors:** Tianyu Guan, Nan Zhang

**Affiliations:** Centre of Micro/Nano Manufacturing Technology (MNMT-Dublin), School of Mechanical and Materials Engineering, University College Dublin, Dublin 4, D04 V1W8 Ireland

**Keywords:** Electrodeposition, Ni-based nanocomposite, Lubrication, Friction, Wear

## Abstract

Recently, nanomaterials such as graphene, polytetrafluoroethylene, WS_2_, and MoS_2_ have emerged as pioneering additives and fillers in metal nanocomposite electrodeposition, offering innovative solutions for lubrication and tribological enhancement. Electrodeposition, known for its high efficiency, reliability, operational simplicity, and cost-effectiveness, has become a preferred method for the protection of industrial components from excessive wear or abrasion. In particular, nickel (Ni) matrix composites fabricated via electrodeposition function as an environmentally friendly substitute for coatings such as hard chromium. These Ni-based composites exhibit multifunctional properties, including enhanced hardness, modified surface wettability, improved anti-friction/wear performance, and lubrication properties. This review begins by explaining the principles and mechanisms of electrodeposition, along with the chemical structures and properties of lubricating nanoparticles. It discusses dispersion methodologies of these nanoparticles in the electrolyte solution to address aggregation problems. In addition, it introduces codeposition models for Ni/nanomaterials and examines the key parameters that influence this codeposition process. This review systematically explores the mechanical properties, tribological performance, and surface wettability of resulting Ni-based nanocomposites, along with their potential applications and practical advantages. Finally, it discusses the opportunities and challenges associated with nanomaterial-enhanced metal composites, aiming to introduce new avenues for their utilization in electrodeposition.

## Highlights

1. Nano-sized graphene, MoS₂/WS_2_ and PTFE enhance the durability and lubrication of Ni-based nanocomposites.
2. Pretreatment of nanoparticles is crucial for enhancing mechanical and tribological performance of nanocomposites.
3. Particle type/concentration/size, current density, and dispersion methods affect the performance of nanocomposites.

## Introduction

Electrodeposition is a “one-step” method for the development of metal matrix nanocomposites (MMCs), which mostly involves electroplating to create a coating on another substrate or electroforming to develop an individual composite [1–3]. Through electrodeposition, the structures and properties of the deposits can be tailored by adjusting the composition of the bath and processing parameters. Consequently, the grain size [4], surface morphology [5], surface roughness (Sa) [6], hardness [7], surface wettability [8] and deposit thickness [9] can be feasibly controlled. Compared with other MMC fabrication methods, such as chemical vapor deposition and mechanical alloying, electrodeposition offers more distinct advantages, including precise current modulation [10], low processing temperature [11], low cost [12], low energy consumption [13], and high scalability for industrial applications [14]. In addition, electrodeposition allows for precise control of coating thickness and nanoparticle incorporation, particularly in the case of complex geometries [15], making it ideal for the fabrication of thick, complex, and high-precision composite coatings/structures on various substrates.

Composite electrodeposition, also called metal particle codeposition, originated in 1928 in a study of Cu–graphite composite coating [16]. In the 1960s, Ni/SiC composite coatings were first used for components of Wankel engines. During the 1970s, there was a rising interest in a wider variety of nickel coatings incorporating materials such as Al₂O₃, graphite, and polymer particles (e.g., polytetrafluoroethylene (PTFE)), particularly for applications in lubrication and corrosion protection [1]. Composite plating has now developed into a distinct subfield of material finishing/processing/manufacturing. This review highlights the recent advancements, particularly over the past decade, with a focus on specialized, Ni-based nanocomposites incorporating solid-state lubricants (e.g., graphene, MoS₂, WS₂, PTFE).

**Fig. 1:**
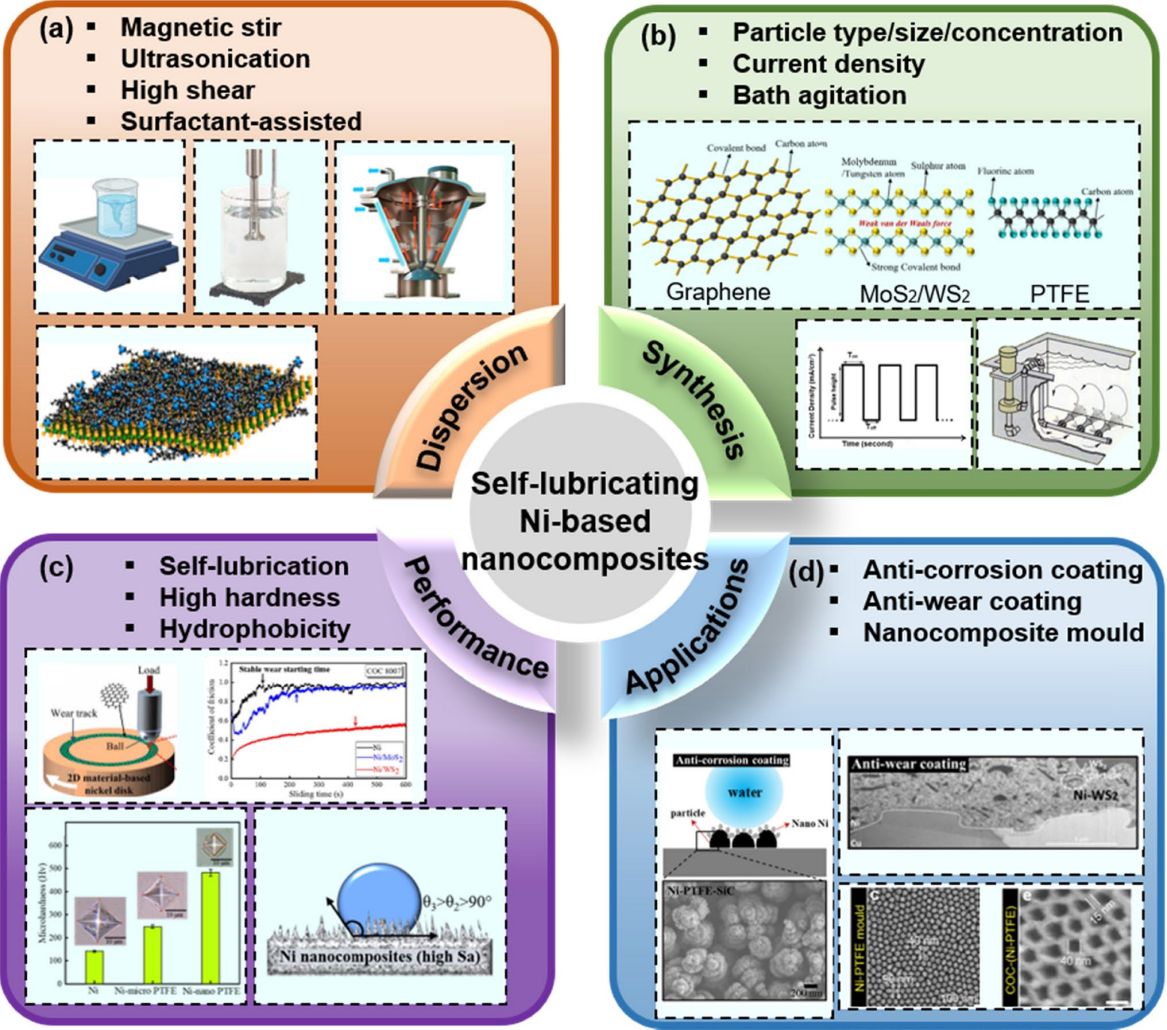
Ni-based lubricating nanocomposites: commonly used dispersion methods **a**, synthesis parameters **b**, performance **c**, and applications **d**

Electrodeposited nickel composites offer superior advantages, including enhanced mechanical [17] and tribological performances [18], high corrosion resistance [19], versatile surface-finishing [20, 21], and high thermal stability [22]. They, thus, can be used in diverse fields (Fig. 1), including (i) application as coating onto metal substrates to eliminate the need for conventional and costly heat treatment [23]; (ii) replacement of the traditional chromium (Cr) coatings owing to the innovative amalgamation of properties [24]; (iii) use as protective coating with anti-corrosion [25], anti-friction [26], or anti-wear performance [27]; (iv) use in fuel cell electrodes [28]; and (v) electroforming of nanocomposite mold tools with extended tool lifetime [29–31].

In Ni-based composites, the introduction of diverse micro/nanoparticles considerably influences the performance of the resultant coatings. Notably, Ni nanocomposites deliver superior mechanical and tribological performances versus conventional composites with microscale fillers. This improvement can be attributed to grain refinement, dispersion strengthening, and enhanced surface finish achieved nanoscale fillers. These nanocomposites encompass a range of particles, including polymers (such as PTFE [32]), metal oxides (such as Al_2_O_3_ [33] and TiO_2_ [34]), nitrides (such as Si_3_N_4_ [35]), carbides (such as SiC [36] and WC [37]), carbon-based fillers [38–40] (including graphite [41], graphene oxide [42, 43], and carbon nanotubes [44–46]), and layered two-dimensional (2D) fillers (such as WS_2_ [29] and MoS_2_ [47]). Of these filler materials, layered 2D materials and the polymer PTFE exhibit inherent low-friction characteristics and are commonly employed as lubricants. When doped with a Ni matrix, they contribute to reducing friction and enhancing wear resistance (Fig. 1).

This review primarily delves into the advancement of electrodeposited Ni-based nanocomposites reinforced with nano-sized lubricating particles, including PTFE, graphene, MoS_2_, and WS_2_. Uniformly dispersing these nanoparticles during codeposition is critical to enhancing performance, as aggregated particles can induce porous structures, roughening the nanocomposites and compromising their mechanical and tribological properties. Despite its importance, the pretreatment of nanoparticles is underappreciated in the literature, a topic thoroughly elucidated in this review regarding its influence on deposit performance. Moreover, this review extensively discusses three critical factors directly influencing the microstructure and performance of Ni-based nanocomposites: particle characteristics, current density, and dispersion methods during codeposition. It provides insights into the enhancement of mechanical properties, modification of Sa and surface wettability, and tribological performance, friction, wear, and lubrication of these nanocomposites. Furthermore, the lubrication mechanism of such nanocomposites is revealed through analysis of their friction and wear behaviors against various counterpart materials. Finally, this review outlines applications across diverse fields, highlights neglected challenges, and proposes future perspectives for the advancement of Ni-based lubricating nanocomposites.

## Fundamentals of the Electrodeposition

### Electrodeposition

Electroplating of Ni means coating a metal with a thin layer of Ni, which is widely used for surface finishing processes because of its corrosion resistance or electrical conductivity [20]. In electroplating, the Ni coating forms a metallurgical bond with the substrate, becoming an integral part of the surface. Unlike electroplating, Ni electroforming involves the “non-adherent” deposition of Ni onto a mandrel; the mandrel is then separated from the deposited Ni upon removal from the plating solution [48]. The applications of electroforming are diverse, including the creation of molds, dies, meshes, and other critical components vital to operations in aerospace, electronics, and automotive industries [49–52]. During the electrodeposition process, a pair of metal electrodes is submerged into the electrolyte solution. Subsequently, an external electric field is employed to deposit the desired metal onto the designated working electrode, commonly referred to the cathode. Adjusting the electrode potential, current density, and plating time enables precise control of the thickness of the metal deposits (Fig. 1).

### Principles and Mechanisms

**Fig. 2:**
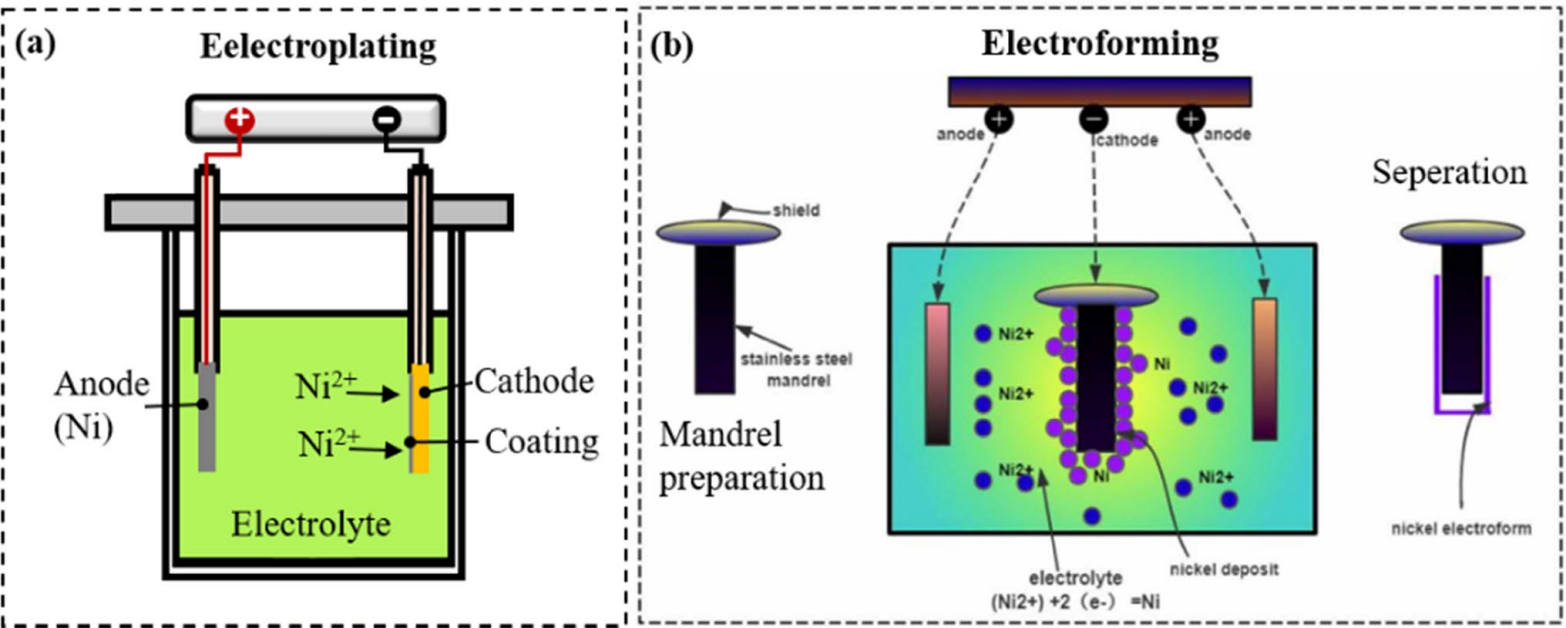
Basic principle of electroplating (**a**) and electroforming (**b**) [50]

In electrodeposition, an external current power source supplies a one-way current to the electrode system. This current flow corresponds to electron movement in the circuit. In the electrolyte, electrical current manifests as ion flow, with the negative ions (anions) traveling toward the anode and the positive ions (cations) toward the cathode because of the external current field. As shown in Fig. 2, nickel ions (Ni^2+^) migrate toward the cathode upon application of direct current (DC). Subsequently, these ions undergo reduction to individual atoms and deposit atom by atom onto the cathode’s surface [53]. During the electroplating process, the Ni deposits remain on the mandrel as a protective coating and become a part of it (Fig. 2a). On the other hand, such Ni deposits can be separated from the mandrel after a certain thickness is achieved, which is a feature of an electroforming procedure [50]. The average thickness (µm) of nickel deposits can be estimated using the following equation, which has been modified as per Faraday’s laws [20]:

$$ {\text{Thickness}} = \frac{m*100}{{d*A}} = 109.5*\frac{aIt}{{8.097*A}} = 12.294*\frac{aIt}{A} $$

where *I* (A) denotes applied current, *A* represents deposit area (dm^2^), the ratio *I/A* (A/dm^2^) denotes current density, *t* is electrodeposition time (min), and *a* is current efficiency ratio (*a* = 1 when cathode efficiency is 100%). One can see that the thickness of nickel deposits depends on current density and electrodeposition time.

### Nanocomposite Electrodeposition

**Fig. 3:**
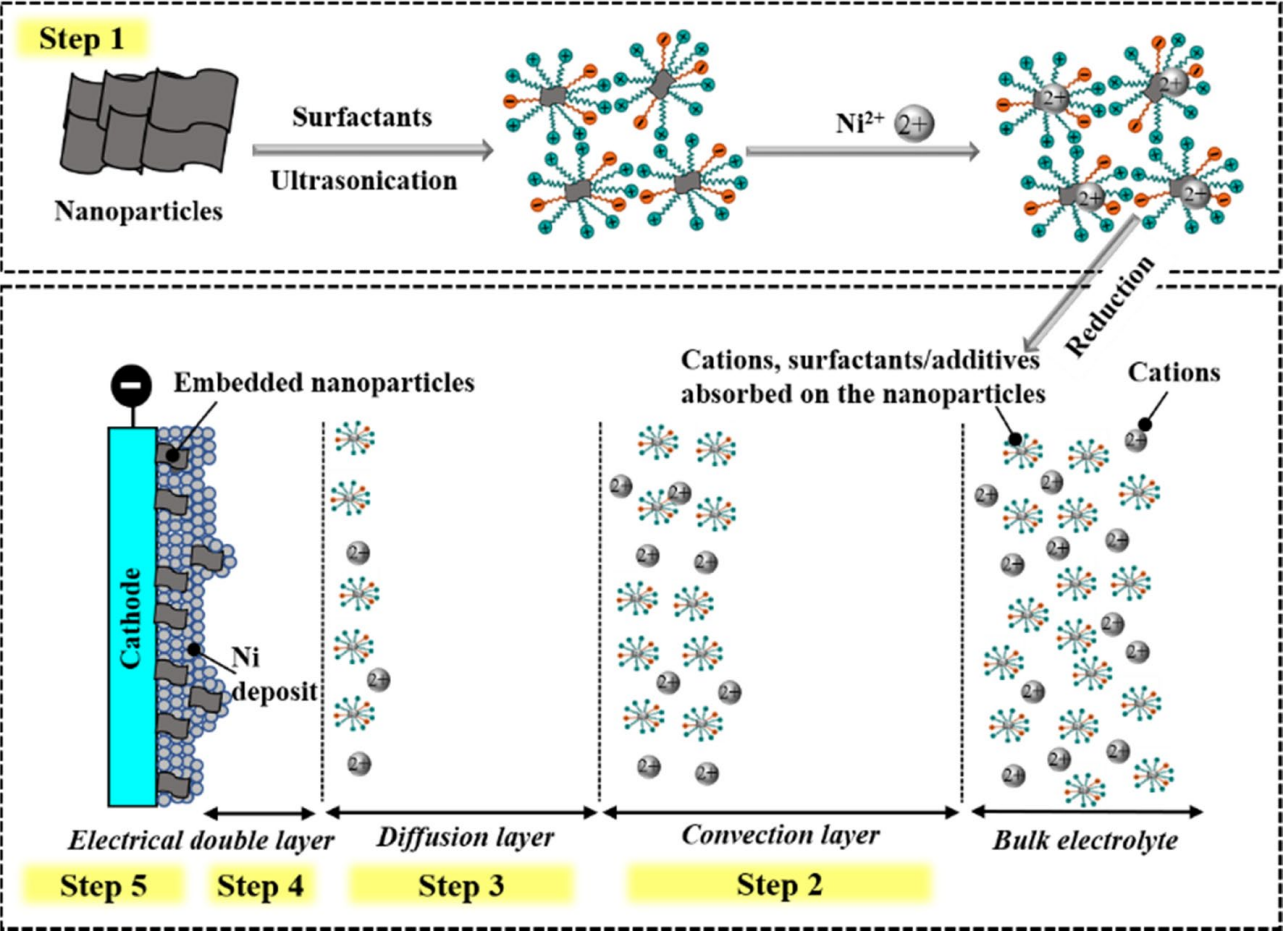
Process of nanoparticle codeposition onto the metal matrix: step 1. ionic cloud formation around particles; step 2. particle convection toward the cathode; step 3. particle diffusion through the hydrodynamic boundary layer; step 4. particle diffusion through the concentration boundary layer; step 5. particle adsorption onto the cathode surface

Codeposition of metal and nanoparticles typically involves two main processes: dispersing nanoparticles in the electrolyte and their electrophoretic migration [54]. Many models have been proposed to explain particle codeposition, as shown in Table 1. In 1972, Guglielmi described the kinetics of codepositing inert particles with nickel as a two-step process: initially, the particles loosely adsorbed, achieving equilibrium with the suspended particles; this was followed by irreversible adsorption. During codeposition, the transport behavior of these particles was observed to be influenced by the molecular and ionic structures of the adsorbed layer onto the particle surface rather than by the electrolyte solution’s composition [55]. Hwang et al. and Bercot et al. optimized the codeposition model based on Guglielmi’s model, as shown in Table 1. However, the adaptability of these existing models requires validation. Figure 3 shows a codeposition process, which typically includes five sequential steps: (1) formation of ionic clouds around particles, (2) particle convection toward the cathode, (3) particle diffusion through the hydrodynamic boundary layer, (4) particle diffusion through the concentration boundary layer, and (5) particle adsorption on the cathode’s surface, resulting in their deposition onto the metal deposit.

**Table 1:** Existing models elucidating the codeposition mechanism of metal matrix and particles

Model	Year	Particles and metal matrix	Characteristics and assumptions	References
Guglielmi	1972	TiO_2_ (1 µm) /SiC (2 µm) in Ni matrix	In the initial stage, the particles undergo loose adsorption, establishing equilibrium with the suspended particles. Subsequently, in the second stage, the particles undergo irreversible adsorption. This model did not consider fluid dynamic conditions	[55]
Celis, Roos and buelens	1987	Al_2_O_3_ (50 nm) in Cu/Au matrix	This method employs probability concepts to quantify the integration of particles at a given current density. Particle transport correlates directly with ion transport towards the working electrode. Five stages were proposed for the codeposition of inert particles	[58]
Fransaer, celis and ross	1992	PS (11 µm) in Cu matrix; SiC (10 nm-10 µm) in Ni matrix	Employs trajectory analysis to elucidate the codeposition process of non-Brownian particles, including the reduction of metal ions (as elucidated by the Butler-Volmer expression) and the codeposition of particles (as explicated through the trajectory expression)	[59]
Hwang and hwang	1993	SiC (3 µm) in Co matrix	Improved based on Guglielmi’s model. Highlighted the significance of current density in particle codeposition. Codeposition rates are closely linked to the reduction of ions adsorbed on particle surfaces	[60]
Vereecken, shao and searson	2002	Al_2_O_3_ (300 nm) in Ni matrix	Particle transportation to the surface is controlled by convective-diffusion, accounting for the influence of particle gravity and hydrodynamics at varying current densities. Gravitational force becomes relevant when the particle size is smaller than the diffusion layer thickness	[56]
Bercot, Pena-Munoz and Pagetti	2002	PTFE (500 nm) in Ni matrix	Further improved based on Guglielmi’s model. Hydrodynamic conditions (bath agitation) are considered by using a mathematical model	[57]

Currently, most of the composite electrodeposition models are based on micro-scale particles, with only a few addressing nano-scale particles, such as those proposed by Vereecken et al. [56] and Berçot et al. [57]. In particular, Vereecken et al. observed that the influence of forces may notably differ depending on particle size. In their study, the gravitational force was of great importance in the codeposition of 300-nm Al_2_O_3_ in the Ni matrix, whereas this force was negligible in the case of codepositing 50-nm nanoparticles [56]. Furthermore, they discovered that the transport of nano-scale particles was primarily driven by convective diffusion, while convection and gravitational forces were observed to be more important for micro-scale particles. Their study also showed that the rate at which nanoparticles were entrapped in the growing composite was influenced by the composite growth rate, which, in turn, was observed to be controlled by the deposition rate.

In contrast, Berçot et al. [57] developed a mathematical model focusing on the mechanical stirring involved in the codeposition of Ni and PTFE nanoparticles. This model allowed for precise predictions of the volume content of incorporated PTFE on varying agitation rates and nanoparticle concentrations. Despite the advances made by these models, further validation and refinement are needed to fully account for the unique behaviors of nano-sized particles in codeposition processes.

## Characteristics and Pretreatment of Nanomaterials

### Characteristics of Nanomaterials

For Ni-based composites, incorporating nano-sized particles results in unique lubrication properties in comparison with conventional micro-sized composites. Nanomaterial fillers are extensively used as promising reinforcements to fabricate Ni matrix nanocomposites exhibiting enhanced mechanical and tribological performances. Nano-sized carbides (SiC [61], TiC [62], WC [63]), metal oxides (Al_2_O_3_ [33, 64, 65], TiO_2_ [34], Cr_2_O_3_ [66]), 2D materials (MoS_2_ [67, 68]/WS_2_ [69, 70], graphene [71], boron nitride [72]) and the fluoropolymer PTFE [73] are commonly used as enhancement fillers. Of these nanoparticles, 2D materials and PTFE have been proven to afford the nanocomposites with remarkable lubrication properties, represented by notably reduced coefficient of friction (COF) and greatly enhanced wear resistance. Notably, the type of lubricating nanoparticles in Ni-based nanocomposites considerably influences their mechanical, tribological, and surface properties.

#### Graphene

**Fig. 4:**
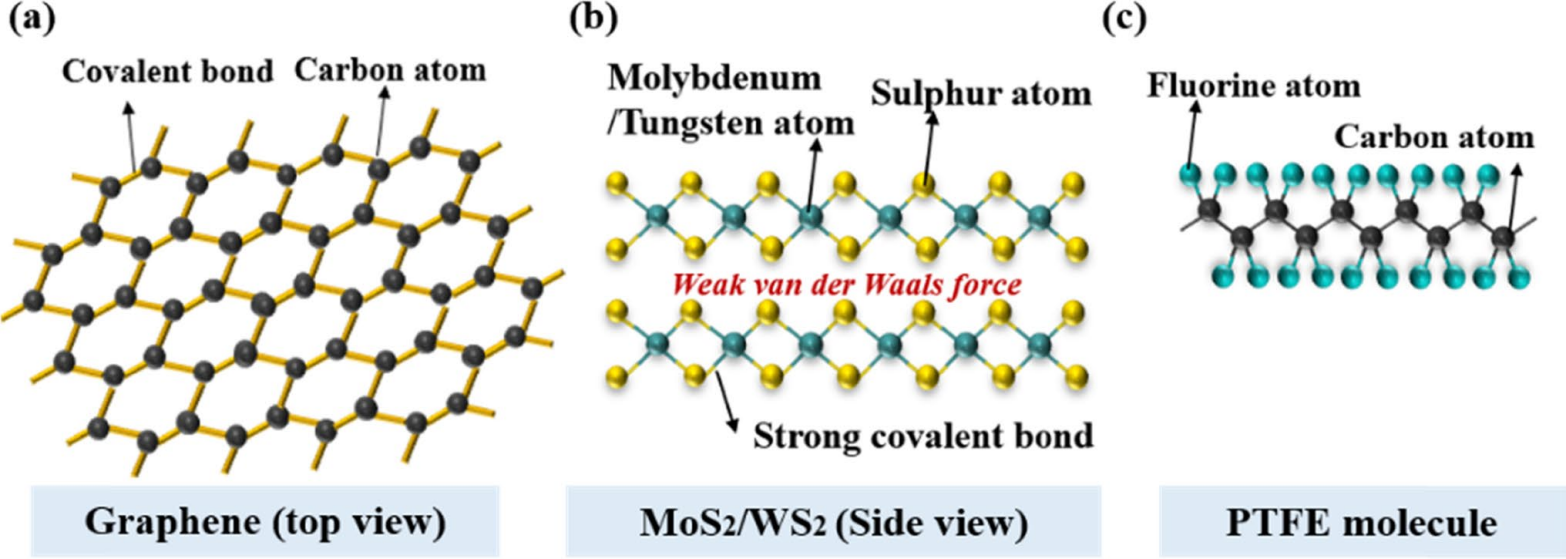
Chemical structures of graphene **a**, MoS_2_/WS_2_ **b**, and PTFE **c**

Graphene is a single-atomic carbon sheet forming a 2D structure with sp^2^ bonding, wherein carbon atoms are densely arranged in a honeycomb lattice (see Fig. 4a). The bonding between carbon atoms within each layer is strong, while the van der Waals interactions between the layers are weak [74]. With a large surface area that closely interacts with the metal matrix, graphene also exhibits a high elastic modulus and high yield strength, along with favorable electrical and thermal conductivities [75, 76]. Graphene serves as a solid lubricant and affords nanocomposites with self-lubricating properties [28, 34]. Introducing graphene into the metal matrix notably enhances the friction and wear characteristics of Ni nanocomposites compared with pure Ni matrix itself [35]. The inclusion of graphene nanosheets in nanocomposites reduces damage accumulation during the wear, thereby notably decreasing the wear rate. Ni–graphene nanocomposites were found to have greatly enhanced hardness and wear resistance in comparison with pure Ni [77]. The thin layer structure of graphene was observed to form a protective deposit between friction surfaces, reducing oxidation wear and furrowing at elevated temperatures, thereby enhancing the wear resistance and friction reduction of the matrix [78].

Graphene nanosheets (with an average thickness of 5–100 nm) are commonly used for the fabrication of metal matrix nanocomposites for industrial applications such as aerospace and automotive [74]. In such applications, graphene-embedded nanocomposites provide remarkable strength, ductility, and firmness as a protective covering, enhancing the endurance of the metal matrix to wear and corrosion. In addition, graphene-embedded metal matrixes also find applications in catalysis [79], surface engineering [80], and energy storage [81]. Nevertheless, the feasibility of employing graphene nanosheets is constrained by their expensive synthesis methods [82], poor solubility and dispersibility [71], and tendency to aggregate [83]. Such problems can be overcome by surface functionalization or surface modification of graphene nanosheets [82, 84].

#### Transition Metal Dichalcogenides (MoS_2_/WS_2_)

Transition metal dichalcogenides (TMDs) comprise a transition metal atom (such as Mo or W) and a chalcogen atom (such as S, Se, or Te) [85], with notable examples being MoS_2_, MoSe_2_, WS_2_, and WSe_2_. As shown in Fig. 4b, these compounds have a layered structure characterized by strong ionic–covalent bonds within each plane and weak interplanar van der Waals forces [86]. The weak van der Waals interactions between the layers are crucial, yielding minimal shear between individual layers and thus notably minimizing the wear and friction during sliding. Such low interplanar shear makes these TMDs great lubricant fillers for the fabrication of metal-based nanocomposites. However, key limitations associated with TMD-based lubricants are their heightened sensitivity to humidity [87] and surface oxidation under severe wear [88]. For example, WS_2_ can transfer into WO_3_ upon oxidation, as observed from the wear track after sliding [89].

Despite these drawbacks, TMD-based self-lubricating nanocomposites are essential in ultra-high vacuum environments as well as in transportation and space systems, including satellites and launch vehicles [90, 91]. MoS_2_ and WS_2_ have received greatest attention as lubricant fillers used in metal matrixes. MoS_2_ and WS_2_ nanoparticles show size-dependent performance [92]. For example, the MoS_2_/WS_2_ nanosheet may have dimensions higher than 100 nm, while the nanodots have dimensions smaller than 5 nm [92]. Incorporation of such nanosheets of different sizes would alter the mechanical and tribological performances, as well as the surface properties, of the resulting nanocomposites [14, 29]. MoS_2_ and WS_2_ nanosheets were observed to offer low COFs, low wear rate, and high corrosion resistance of Ni composites [93, 94], with WS_2_ providing high microhardness and wear resistance to the deposits because of its inherent high hardness [14, 51].

#### PTFE

PTFE’s molecular structure is shown in Fig. 4c. Its molecular formulation incorporates the robust C–F bond, represented by the molecular formula [(CF_2_–CF_2_)_n_]. The high molecular weight of PTFE is attributed to the strong bonds formed between fluorine atoms, contributing to its semicrystalline nature [95]. The strong C–F bond in PTFE, with a bond strength of ~ 460 kJ/mol, prevents itself from reacting with other compounds [96]. This inert behavior can be ascribed to the tightly bonded carbon backbone and closely packed fluorine atoms within the molecular structure. The stability arising from this strong bond imparts durability to PTFE and makes it resistant to chemical reactions [97].

Notably, the properties of PTFE strongly depend on its molecular weight. PTFE, with its high molecular weight, is generally chemically inert [97]. In addition, PTFE has an extremely low COF and remarkable thermal stability (> 400 °C) [97]. Of its properties, hydrophobicity and anti-adhesion merit the greatest attention. Hydrophobic surfaces are distinguished by a static water contact angle (WCA) exceeding 90°, indicative of their inherent water-repellent nature. Likewise, the contact angle observed on smooth PTFE surfaces is within the range of 108°–114°[98]. Incorporation of such hydrophobic nanoparticles would impart a hydrophobic nature to Ni-based nanocomposites, as confirmed by many studies [32, 99–101]. Surface lubrication in PTFE-embedded nanocomposites is typically remarkable because of the presence of highly electronegative fluorine atoms. However, incorporating PTFE nanoparticles into the Ni matrix may result in reduced wear resistance, particularly when sliding against a metal counterpart [102]. This can be attributed to the reduced hardness of Ni–PTFE composites.

### Pretreatment of Nanoparticles

Electrodeposition has become an important method to synthesize nanocomposites with enhanced performance. When codepositing a metal and nanoparticles, the growth of the nanoparticles assumes great importance. This factor critically influences the size distribution of nanoparticles in the solution and the crystal structure of the resulting nanocomposites, in turn affecting the mechanical and tribological performances, as well as the surface properties, of the metal composites.

For example, Van Hau et al. observed that the size of graphene nanoplatelets (GNPs) notably affected the microhardness of Ni–GNP composites. Their study revealed that smaller GNPs (~ 120 nm) could intercalate within the crystal nucleation sites of Ni, facilitating grain refinement and dispersion strengthening, which greatly enhanced the microhardness of the nanocomposites. In contrast, larger GNPs (250–600 nm) were unable to intercalate into the Ni crystal structure, resulting in lower microhardness [103]. Thus, to improve the mechanical performance of Ni-based nanocomposites, disintegrating nanoparticles via surface modification and effective dispersion in the electrolyte solution become a necessary pretreatment procedure before electrodeposition. In this section, we provide a comprehensive overview of dispersion methods, particularly in the divalent Ni-based electrolyte solution (Table 2). This includes a detailed exploration of surface modification through the use of surfactants [104], dispersion by high shear mixing [105, 106], ultrasonic treatment [14], and other relevant techniques.

**Table 2:** Dispersion methodologies for lubricating nanoparticles in aqueous and divalent Ni-based electrolyte solutions

Particle type	Particle size (nm)	Dispersion media	Disperse method	Outcome	Advantages/Disadvantages	Friction test condition and tribological performance	References
Graphene (0.1 g/L)	NA	NiSO_4_·6H_2_O (nickel sulfate)	Sodium dodecyl sulfate (SDS, 0.2 g/L); magnetic stirring (200 rpm)	With SDS, a smoother surface is generated. Enhanced microhardness and wear performance	It is easy to use but hard to scale up	Alumina ball, 10 N, 0.1 m/s, COF was 0.45 for Ni, decreased to 0.39 for Ni-Graphene-SDS	[120]
Graphene (0.1–0.4 g/L)	NA	NiSO_4_·6H_2_O	Sodium dodecyl benzenesulfonate (SDBS) (0.05 g); magnetic stirring for 2 h (750 rpm); ultrasonic agitation for 1 h	More compact surface morphology and higher hardness were generated with the increase in graphene content	It is easy to use but requires a long treatment time and is hard to scale up	Si_3_N_4_ ball, 1-3 N, 0.1 m/s, COF was 0.9 for Ni, reduced to 0.6 for Ni-Graphene (0.4 g/L)	[121]
Graphene oxide (GO)	0.6–1 nm thickness, 0.5–5 µm particle size	Deionized (DI) water	Polyethylene glycol trimethylnonyl ether (0.1vol%); ultrasonic treatment until the solubility of GO is high in DI water	Nanocrystalline Ni-GO composite was developed. GO transformed into reduced GO during plating. Enhanced microhardness and wear resistance	It has good dispersion, but it is hard to control the surface charge	Steel ball, 10 N, 200 rpm. COF was 0.457 for Ni, which declined to 0.247 (Ni-GO)	[122]
GO (0.4 wt.%)	Several layers, lateral dimensions 100 nm- 200 µm	Nickel sulphamate solution	SDS (0.8 g/L)/ CTAB (0.8 g/L)/ PEG (0.8 g/L); mechanical stir for 20 h (500 rpm); ultrasonic probe for 20 min	Dispersion stability: PEG > CTAB > SDS. More stable dispersion increased the weight percentage of GO in the Ni matrix, further affecting the composite performance	It is easy to use but requires a long treatment time and is hard to scale up	Stainless steel ball, 5 N, 31.8 rpm. COF was 0.55 for Ni, reduced to 0.15 for Ni-GO-PEG	[123]
Graphene (0.2 g/L)	Interlayer distance of 0.4 nm	NiSO_4_·6H_2_O	SDS (0–0.4 g/L); magnetic stirring (300 rpm)	SDS roughened the surface of the nanocomposite coating. Bulge morphologies were detected on Ni-GO (SDS 0.4 g/L). Higher carbon content and better mechanical performance due to higher SDS concentrations	It is easy to use but has poor dispersion and is hard to scale up	NA	[124]
WS_2_ (0.1 g/L)	Lateral dimensions: 200–300 nm	Nickel sulphamate solution	CTAB (1.0 g/L) with SDS (0.3 g/L); intermittent ultrasonication for 20 min after every 15 min	Particle size was reduced to 106 nm, with a polydispersity index (PDI) of 0.20. Microhardness, surface hydrophobicity and wear resistance were increased	It has good dispersion and is good to scale up	PMMA pin/COC 8007 pin, 1 N, 31.8 rpm. COF decreased from 0.91 (Ni) to 0.45 for Ni-WS_2_, against PMMA, from 0.93 for Ni to 0.47 for Ni-WS_2_ against COC	[14]
WS_2_ (0.1 g/L)	100–300 nm	Nickel sulphamate solution	CTAB and SDS (both at 1.0 g/L); ultrasonication for 15 min	Improved dispersibility in Ni matrix. Enhanced microhardness, surface hydrophobicity and wear resistance	It is easy to use and has good dispersion, but it is hard to scale up	PMMA pin, 1 N, 31.8 rpm. COF declined from 0.75 (Ni) to 0.31 (Ni-WS_2_)	[29]
WS_2_ (0.14 g/L)	1 µm for lateral dimension and 0.7 nm for thickness	Nickel sulphamate solution	CTAB (0.1 g/L); ultrasonic probe for 20 min; magnetic stirring during deposition (720 rpm)	WS_2_ (0.14 g/L) showed best dispersibility in nanocomposites. Enhanced microhardness and wear resistance	It has good dispersion but is hard to scale up	Stainless steel ball, 1 N, 31.8 rpm. COF reduced from 0.65 (Ni) to 0.18 (Ni-WS_2_)	[51]
WS_2_ (0.3 g/L)	NA	NiSO_4_·6H_2_O	SDS, ultrasonication for 60 min	The Ni-W-WS_2_ exhibits a compact surface structure, enhanced microhardness of 834 Hv, and superhydrophobic characteristics with a WCA of 154.4°	It has good dispersion but requires a long treatment time; it could cause local overheating of the treated solution and is hard to scale up	Si_3_N_4_ ball, 20 N, COF decreased by 47% compared with Ni-W coating to 0.14 for Ni-W-WS_2_	[125]
WS_2_ (1.0 g/L)	NA	NiSO_4_·6H_2_O	PEG (0.2 g/L), SDS (0.04 g/L), magnetic stirring (400 rpm)	Twice higher microhardness	It is easy to use but requires a long treatment time and is hard to scale up	Steel ball, 1 N, 143 rpm. COF decreased from ~ 0.9 to ~ 0.1 for Ni-WS_2_	[126]
MoS_2_ (1.0–2.0 g/L)	1–4 µm for lateral dimensions	NiSO_4_·6H_2_O	Dodecyltrimethylammonium Bromide (DTAB) (0.028 g/L), mechanical stirring for 1 h (120 rpm) and ultrasonication for 1 h	Ultrasonic-assisted codeposition can generate a more uniform Ni-MoS_2_ coating with higher hardness	It has good dispersion. Long treatment time could cause local overheating of the treated solution. It is hard to scale up	15 GCr steel ball, 320 g, 150 t/m. COF was ~ 0.9 for Ni, decreasing to ~ 0.15 for Ni-MoS_2_ with the use of 1.0 g/L MoS_2_	[68]
MoS_2_ (0.1 g/L)	Lateral dimensions: 200–300 nm	Nickel sulphamate solution	CTAB (1.0 g/L) with SDS (0.4 g/L); intermittent ultrasonication for 20 min after every 25 min	Particle size was reduced to 104 nm, with a PDI of 0.24. Increased hardness and surface hydrophobicity	It is easy to use and has good dispersion, but it is hard to scale up	PMMA pin/COC 8007 pin, 1 N, 31.8 rpm. COF decreased from 0.91 (Ni) to 0.88 (Ni-MoS_2_) against PMMA and from 0.93 (Ni) to 0.86 (Ni-MoS_2_) against COC	[14]
MoS_2_ (1–4 g/L)	2–4 μm	NiCl_2_.6H_2_O (nickel chloride)	CTAB (0.1 g/L), ultrasonic treatment for 30 min, agitation (50, 150, 200 rpm)	Increasing the agitation speed of the bath to 150 rpm effectively enhanced the embedding of MoS_2_ and resulted in improved wear resistance. However, agitation speeds beyond 150 rpm caused a decrease in the incorporation of MoS2 particles into the nickel matrix, likely due to excessive turbulence in the bath	It requires moderate treatment time, has good dispersion, and is suitable to scale up	Steel pin, 1.6 N, 62 rpm. COF decreased from 0.35 to 0.08	[127]
MoS_2_ (3–20 g/L)	1–4 μm	NiCl_2_.6H_2_O	CTAB (0.1 g/L), ultrasonic bath	A compact structure with a high MoS_2_ content was achieved for the Ni–P-MoS_2_ composite, ensuring a robust, low-friction coating	It is easy to use and has good dispersion. However, it is hard to scale up due to the limited space of the ultrasonic bath	Steel ball, 14N, 1 Hz. COF reduced from 0.45 to 0.05 for Ni–P-MoS_2_	[47]
MoS_2_ (2 g/L)	1–2 μm	NiSO_4_·6H_2_O	CTAB (0.1 g/L), high shear mixing (8000 rev/min)	A narrow particle-size distribution (200 nm-1.9 μm) together with reduced particle agglomerates (~ 1150 nm) was achieved	It is easy to use and has good dispersion, but it is hard to scale up due to the increased volume of the electrolyte	Steel cylinder, 20N, 1 Hz. COF decreased from 0.16 to 0.08	[105]
PTFE (0–40 g/L)	0.6–8 μm	NiSO_4_·6H_2_O	CTAB (0.033 g/g PTFE), ultrasonic treatment for 1 h	A superhydrophobic surface (WCA 152°) was achieved. Small particles were distributed more uniformly than large particles in the Ni matrix. The microhardness of the coatings showed notable variation depending on the specific local PTFE content	It has good dispersion but requires a long treatment time, which could cause local overheating of the treated solution. It is hard to scale up	NA	[99]
PTFE (0.5 g/L)	100–300 nm; 1–3 μm	Nickel sulphamate solution	CTAB (1.0 g/L) with SDS (0.4 g/L), magnetic stir for 30 min, followed by ultrasonication for 20 min	Particle sizes of PTFE nanoparticles/microparticles were reduced to ~ 106 nm (PDI 22.91%) and ~ 1081 nm (PDI 28.11%), respectively. Microhardness, surface hydrophobicity, and wear resistance of Ni-PTFE nanocomposites were much higher compared with microcomposites	It is easy to use and has good dispersion, but it requires a long treatment time and is hard to scale up	PMMA pin, 1 N, 31.8 rpm. COF decreased from 0.46 (Ni) to 0.41 (Ni-PTFE microcomposites) and further to 0.22 (Ni-PTFE nanocomposites)	[114]
PTFE (15 g/L)	NA	Ni(SO_3_NH_2_)_2_·4H_2_O	C_21_F_17_H_20_O_3_N_2_SI (3 mL/L)	Cracks of ~ 20 nm were observed near the PTFE particles due to high internal stress. The sidewall roughness is low (~ 24 nm)	Dispersion remains unknown	8.6% Cr steel ball, 500 mN, 20 Hz. COF decreased with the increase of PTFE content, specifically from 0.4 for Ni to 0.2 for Ni-PTFE composite	[128]
PTFE (5–20 g/L)	NA	Nickel sulphate	CTAB (0.5 g/L); mechanical stirring for 24 h (600 rpm)	PTFE particles were evenly distributed within Ni-W composites, resulting in moderate hardness, higher corrosion resistance, smooth surface, and hydrophobicity (CA 109.9°)	It requires a long treatment time and is hard to scale up	Scratch test was applied for COF, 5 N, 0.2 m/s. The COF reduced from 0.78 for Ni to 0.58 for Ni-W and decreased significantly to ~ 0.2 for Ni-W-PTFE	[129]
PTFE (50 mL/L)	NA	Nickel sulphate	Magnetic stir during plating	Ni-Co-PTFE exhibits superior wear resistance and hardness compared to Ni-Co and Ni under various loads and sliding velocities	It has insufficient agitation and is hard to scale up	COF was consistently lower for Ni-Co-PTFE compared to Ni-Co and Ni under various loads and sliding velocities, attributed to the more uniform distribution of the PTFE lubricating film across the composite material	[130]

Surfactant-assisted dispersion is the most commonly used method for exfoliation of nanosheets in aqueous media, involving several key processes: (1) Reduction of surface tension: Surfactants lower the interfacial tension between the nanoparticles and solution, making it easier for the particles to be evenly distributed within the medium and reducing the energy required for dispersion [107]. (2) Adsorption on nanoparticle surface: Surfactants have amphiphilic structures with a hydrophilic head and a hydrophobic tail. When a surfactant is added to the nanoparticle suspension, its molecules adsorb onto the nanoparticle surfaces. The hydrophobic tail interacts with the surface of the nanoparticle, while the hydrophilic head extends outward into the solvent, increasing the solubility of nanoparticles [108]. (3) Steric and electrostatic stabilization: In steric stabilization, surfactant molecules form a physical barrier around nanoparticles, preventing the nanoparticles from coming too close to each other and forming aggregates. This occurs when bulky surfactant molecules or polymer chains create spatial separation between particles [109]. For charged surfactants, the hydrophilic heads introduce a charge on the nanoparticle surface, resulting in electrostatic repulsion between similarly charged particles. This prevents these particles from coming together and forming larger aggregates [14].

**Fig. 5:**
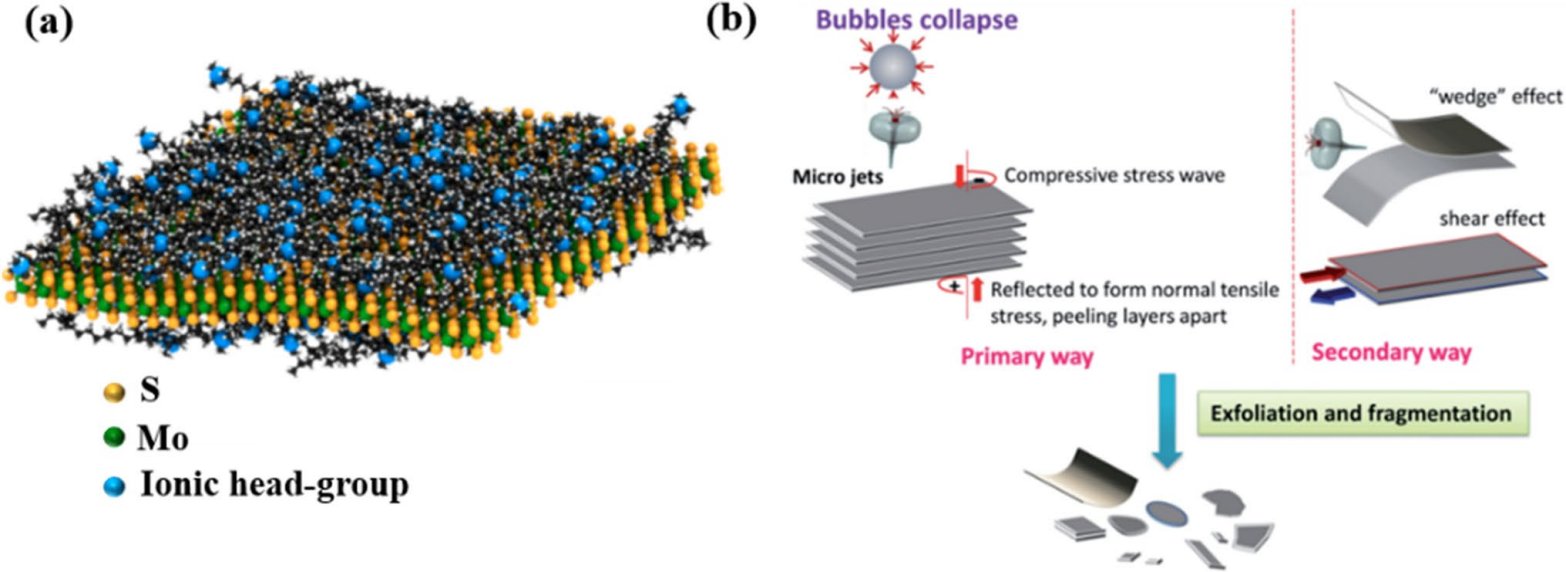
Mechanism of using ionic surfactants for dispersing MoS_2_ nanosheets in aqueous solution **a** [104] and using sonication for particle exfoliation **b** [117]

As shown in Fig. 5a, Gupta et al. observed that MoS_2_ nanosheets can be stably dispersed in aqueous solutions via sonication and ionic surfactant modification. The stability of the dispersion was maintained via electrostatic repulsion between the nanosheets. Moreover, surface charge on the MoS_2_ nanosheets could be controlled via the selection of proper surfactants to be modified as either positive or negative [104]. They also observed rapid exchange between bound and free surfactant chains, revealing that these surfactant chains were arranged in a random fashion, lying flat on the MoS_2_ surface as a monolayer arrangement (Fig. 5a). This result confirmed that the surfactant chains exhibited weak binding to the nanosheets, exposing the charged headgroups [104]. This elucidated the feasibility of modifying the nanosheets with either a positive or negative charge in an aqueous solution. In Poorsargol’s research, graphene exhibited higher adsorption capacity for pure sodium dodecyl sulfate (SDS) and an anionic-rich mixture in comparison with pure cetyltrimethylammonium bromide (CTAB) and a cationic-rich mixture, attributed to the smaller size of SDS. Interestingly, the dispersion of graphene nanosheets remained stable even at a lower total concentration of surfactant mixture than that when each surfactant was used alone, suggesting a synergistic benefit in catanionic combinations [110]. This underscored the complex interplay of surfactant types and concentrations in optimizing the dispersion characteristics of graphene nanosheets.

Notably, the dispersion of lubricating nanoparticles within the electrolyte solution could be different. Our group systematically investigated the dispersion stability and surface charge of nanoparticles within the nickel sulfamate solution after surfactant treatment. As for the use of non-ionic surfactants, Zhang et al. discovered that graphene oxide exhibited optimal dispersibility when treated with the non-ionic surfactant polyethylene glycol (PEG), outperforming ionic surfactants such as CTAB and SDS [71]. Guan et al. observed uniform dispersion of hydrophobic WS_2_ nanosheets within the Ni matrix when equal concentrations of the cationic surfactant CTAB and the anionic surfactant SDS were used to modify and disperse them in nickel sulphamate solution. This finding highlighted the effective balance achieved between these surfactants in facilitating the homogeneous integration of WS_2_ nanosheets into the Ni substrate [29]. The synergistic effect of using such surfactant mixtures was also validated by electroforming Ni–Co alloy nanocomposites containing MoS_2_/PTFE nanoparticles [111].

However, for scaling up the fabrication of 4-inch Ni-based nanocomposites, it is essential to control the migration of nanoparticles by adjusting their surface charge. Excessive positive surface charges cause nanoparticles to adhere to the cathode, potentially halting codeposition. Conversely, nanoparticles with a net negative charge are repelled from the cathode, resulting in physical entrapment within the deposits rather than effective incorporation through electrophoresis, thereby reducing nanoparticle content in final nanocomposites [14]. Therefore, for electroforming 4-inch Ni-MoS_2_/WS_2_ nanocomposite molds, a stable (over an extended period) dispersion of MoS_2_/WS_2_ nanosheets in the electrolyte solution was achieved via intermittent ultrasonic treatment alongside the use of cationic-rich surfactant mixtures [14]. Using cationic-rich surfactants was critical to achieving a positive net surface charge with a low zeta potential, enabling effective incorporation of nanoparticles onto the cathode surface via electrophoresis and fabrication of high-quality 4-inch nanocomposite molds.

Other than using surfactants, ultrasonic treatment is also prevalent as a dispersion method implemented before electrodeposition (Table 2). Ultrasonic dispersion of nanoparticles in a solution relies on the application of high-frequency sound waves (> 20 kHz) to break up agglomerates and uniformly disperse the nanoparticles, which is primarily driven by cavitation. As shown in Fig. 5b, exfoliation begins with liquid cavitation during the sonication process. Using graphite flakes as an example, cavitation-induced bubbles surround these flakes. As these bubbles collapse, they create microjets and shock waves, which rapidly compress the surfaces of graphite, generating compressive stress waves propagating throughout the material. Upon reaching the free interface of graphite, these compressive waves produce reflected tensile stress waves within the structure. Consequently, the collapse of numerous microbubbles generates intense tensile stress in the graphite flakes, akin to the action of “sucking disks,” aiding exfoliation. Moreover, an additional mechanism could occur where uneven lateral compressive stress might induce separation between neighboring flakes via a shear effect. Furthermore, the microjets have the potential to split graphite flakes, similar to inserting a wedge into the interlayer. In summary, it is primarily the tensile stress that drives the effective exfoliation of graphite into graphene flakes, establishing a mechanism predominantly governed by normal forces.

Such an ultrasonic exfoliation mechanism can also be applied for dispersing MoS_2_/WS_2_ nanosheets, as evidenced by our recent study [14], in which we achieved small small particle sizes of ~ 100 nm and long-term dispersion during the codeposition process. In addition to applications in graphite flakes and MoS_2_/WS_2_ nanosheets, ultrasonication has also proved effective in reducing the size of PTFE micro/nanoparticles to prevent aggregation during plating, as shown in Table 2.

Ultrasonic treatment parameters, including time, power, and frequency, should be carefully selected to achieve stable nanoparticle dispersions. Typically, the frequency of the ultrasonic probe is fixed (e.g., 20–40 kHz). Frequency influences the size and intensity of cavitation bubbles formed during ultrasonic treatment. Lower frequencies (e.g., 20–40 kHz) can result in high cavitation efficiency, typically employed in nanoparticle dispersions.

Ultrasonic power determines the amount of energy delivered to the dispersion medium. Higher power facilitates cavitation, improving particle separation; however, excessive power may cause particle fragmentation or local overheating, leading to nanoparticle defects [112]. Moderate power levels (e.g., 200–300 W) are typically effective for achieving most particle disintegration. The optimal power should be sufficiently high to achieve deagglomeration but also sufficiently low to avoid damaging the particles or overheating the suspension. Gradual increases in power with close monitoring are recommended to determine the balance between particle disintegration efficiency and the prevention of overheating. For instance, an optimum amplitude of 60% with an ultrasonic power of 500 W was observed to provide the best disintegration effect for MoS_2_/WS_2_ nanosheets with high stability within the electrolyte solution. This is because high ultrasonic power would decrease the effective cavitation intensity, resulting in the cavitation shielding effect and reducing the particle disintegration efficiency.

The duration of ultrasonic treatment directly affects the degree of nanoparticle dispersion. Insufficient time may result in incomplete disintegration, while excessive time can cause particle reagglomeration or even damage to the nanoparticles. Yang et al. conducted an investigation wherein PTFE microparticles, initially averaging 60 µm in size, were successfully disintegrated into nano-sized particles (~ 254 nm) after 4-h ultrasonication in ethanol solutions [113]. In another study, PTFE micro/nanoparticles, with sizes initially in the ranges of 1–3 μm and 100–300 nm, were successfully disintegrated into ~ 1081 and ~ 106 nm particles after 20-min treatment, respectively. After codeposition, the Ni–PTFE nanocomposite mold exhibited a substantially reduced COF and notably enhanced wear resistance against polymer materials. This improvement can be attributed to the smaller crystallite size achieved through the incorporation of nano-sized PTFE particles [114].

Nevertheless, the use of surfactants or ultrasonication also suffers limitations. The incorporation of surfactants, while aiding the dispersion of nanoparticles, poses challenges to the mechanical strength of metal nanocomposites. This is evident from the compromise of the metal matrix’s mechanical properties, for example, reduction in microhardness [44]. Excessive use of surfactants may further render the metal composites overly brittle, a factor often ignored in various studies.

In addition, prolonged application of ultrasonication can overheat the electrolyte solution, thereby affecting its composition [14]. Furthermore, sonication treatment may introduce topological defects into the nanosheets, along with the attachment of oxygen-containing groups to the sheet edges [115, 116]. To address these issues, sonication parameters, including time, power, and frequency, need to be carefully optimized. This optimization should align with practical experimental conditions, accounting for changes in zeta potential, particle size, and polydispersity index of dispersed nanoparticles over plating time.

In addition to parameter optimization, the design of sonication vessels plays a crucial role [117]. The distribution and intensity of ultrasonication-induced cavitation are strongly influenced by the vessel’s size and shape, often resulting in localized cavitation effects [118, 119]. Therefore, vessel size and shape notably affect ultrasonication-assisted nanoparticle dispersion. Consideration should be given to maximizing sonication intensity and ensuring uniform distribution of cavitation. While laboratory-scale nanocomposite plating is commonplace, scaling up such processes remains a notable challenge for practical industrial applications. Addressing the complexities associated with scaling up is pivotal for the successful incorporation of nanocomposite plating in industrial settings.

## Factors Influencing the Codeposition

Successful incorporation of nanoparticles into the Ni matrix is influenced by several factors, including properties of particles, composition of electrolyte, temperature and pH of electrolyte, current density, hydrodynamic conditions, electrode geometry, and bath agitation [1]. Of these, three primary factors are particularly critical to determining the codeposition of lubricating nanoparticles onto the Ni matrix: nanoparticle type and concentration, applied current density (direct or pulsed), and bath agitation.

### Particle Characteristics

**Fig. 6:**
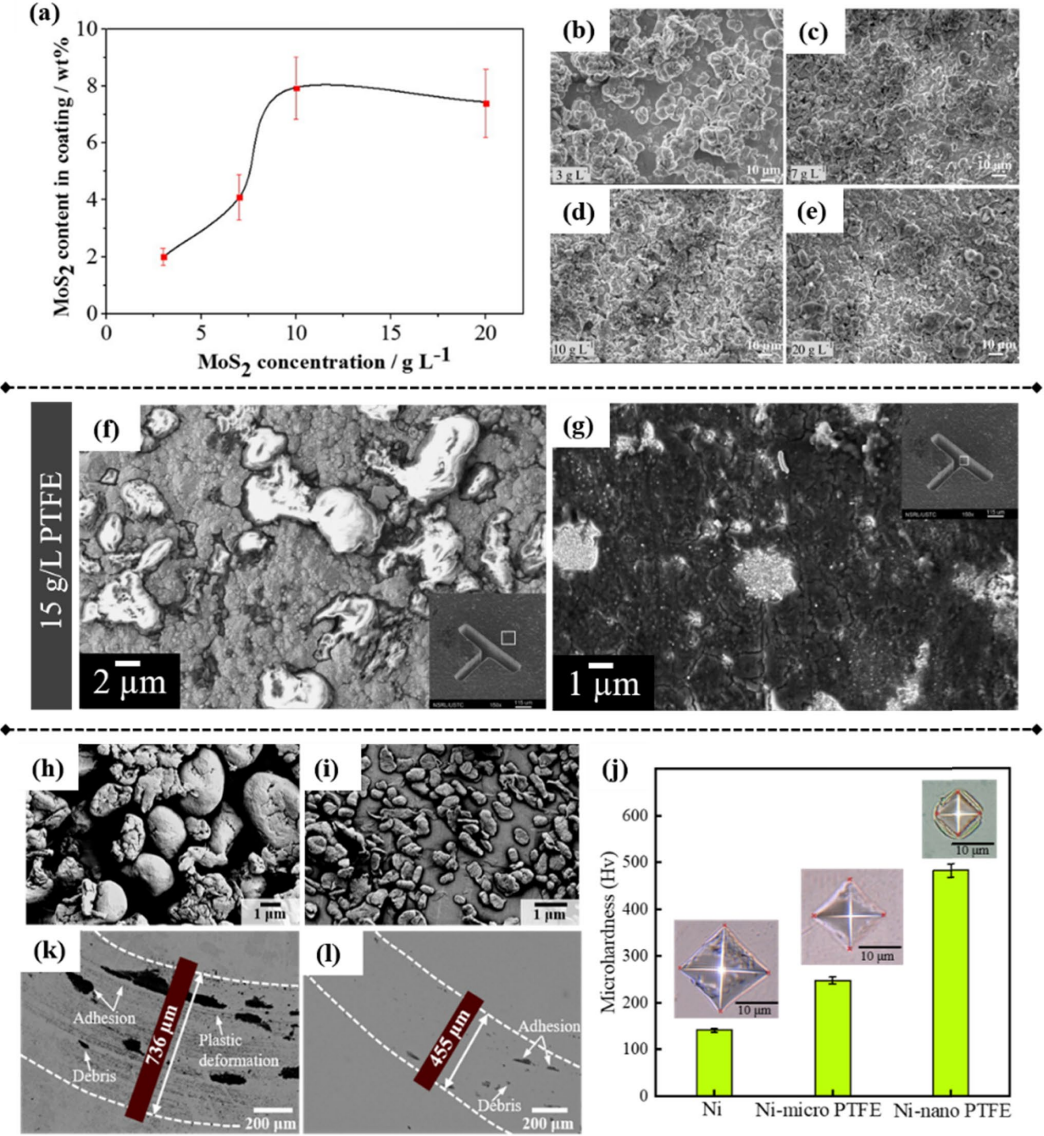
Incorporated MoS_2_ content in Ni–P–MoS_2_ composite coatings at various MoS_2_ bath concentrations **a**. **b**–**e** Surface morphology of Ni–P–MoS_2_ composite coatings with MoS_2_ concentrations of 3, 7, 10, and 20 g/L, respectively. Scale bars: 10 µm [47]. **f**, **g** Ni–PTFE mold created with 15 g/L PTFE particles via electroforming, showing the top surface and sidewall under scanning electron microscopy (SEM), with cracks observed along embedded PTFE particles [128]. **h**, **i** Surface morphology of PTFE micro particles (1–3 µm diameter) and nanoparticles (100–300 nm diameter) [114]. **j** Relationship between hardness and particle size for Ni–PTFE composites [114]. **k**, **l** Wear morphology of Ni–PTFE microcomposites and nanocomposites against PMMA pin after pin-on-disk test [114]

In general, nanoparticle type and concentration are the most notable factors affecting the plating process and the properties of resulting nanocomposites. Particle content in metal composites typically increases with the concentration of particles in the bath until it saturates [1]. He et al. observed that when incorporating MoS_2_ nanoparticles into the Ni–P matrix, particle content in the composites first increased and then stabilized irrespective of further increase in particle loading in the bath (Fig. 6a), attributed to particle aggregation caused by concentration saturation [47]. This observation was further validated by examining the surface morphology of Ni–P–MoS_2_ composites. At low MoS_2_ concentrations, the composite coating exhibited a rough surface with large nodules (Fig. 6b). However, at concentrations of more than 10 g/L, such coating showed a flat surface with a relatively compact structure (Fig. 6d). Further increasing particle concentration to 20 g/L resulted in a rougher surface because of particle aggregation (Fig. 6e).

Nanoparticle aggregation can lead to difficulty in the codeposition process [131]. Zhang et al. investigated how particle type and concentration affected the lubricating properties of Ni-based nanocomposite molds [51]. They demonstrated that WS_2_ notably outperformed MoS_2_ and graphene oxide nanoparticles in reducing the COFs of the nanocomposites against a stainless steel ball. Furthermore, they discovered that a concentration of 0.14 g/L WS_2_ in the electrolyte resulted in optimal lubrication and wear resistance of the resulting Ni nanocomposites, as lower concentrations of WS_2_ were insufficient for adequate lubrication, while higher concentrations led to the formation of aggregates on the Ni matrix and thus compromised its properties.

Other than aggregation, particle size/concentration also affects the internal stress of resultant composites. Guo et al. synthesized a Ni–PTFE composite mold by incrementally adding PTFE particles until reaching a final concentration of 15 g/L. The composite mold demonstrated lower friction against a steel ball, as evidenced by COF decreasing from 0.4 for the Ni mold to 0.2 for the Ni–PTFE mold. However, they detected cracks with dimensions of ~ 20 nm near the embedded PTFE particles, attributed to high internal stress (Fig. 6f and g). Increasing the PTFE content would enhance the lubrication of such composites; however, this would also induce higher internal stress, resulting in nano-sized cracks on the composites.

Utilizing smaller nanoparticles at lower concentrations may offer a viable solution to developing high-performance nanocomposites with low internal stress. Meanwhile, many studies have demonstrated that incorporating nano-sized particles into a metal matrix notably enhances the properties of the resulting composites versus using micro-sized particles [103, 132, 133]. For instance, Ni–PTFE micro/nanocomposite molds were developed by integrating lower concentrations (0.5 g/L) of PTFE micro/nanoparticles into the Ni matrix (Fig. 6h and i). The results indicated that incorporating nano-sized PTFE notably enhanced the microhardness of the composite mold in comparison with those reinforced with micro-sized PTFE particles (Fig. 6j). Consequently, the wear resistance of the Ni–PTFE nanocomposites against a poly(methyl methacrylate) (PMMA) pin was notably improved because of higher hardness and lower surface energy, represented as reduced adhesion- and plastic deformation-induced wear (Fig. 6 k and l). Notably, no cracks were observed in the Ni–PTFE nanocomposite mold, and the Sa and quality were comparable to those of pure Ni mold [114].

Research incorporating graphene [134] and graphene oxide [135] has shown that increasing particle content in the metal matrix can degrade performance, including corrosion resistance. Elevated concentrations often cause nanoparticle agglomeration or uneven particle distribution within the composites.

In addition, the distribution of lubricating nanoparticles within the nanocomposites would also affect the properties of deposits. Uniformly distributed graphene nanoparticles within the Ni matrix were observed to restrain the growth of Ni grains, reducing the plastic deformation of the deposits via grain refinement and dispersion strengthening effect [103]. Similarly, Liu et al. observed that a low-volume fraction of graphene provided high tensile strength and ductility for Ni–graphene nanocomposites; however, this enhancement effect diminished when graphene agglomerated within the high-volume plating bath, proving the importance of uniform distribution of nanoparticles within the nanocomposites [136]. Iacovetta et al. observed that the microhardness of Ni–PTFE composites strongly depended on the local concentration of PTFE particles [99]. Consequently, careful consideration needs to be devoted to the type, size, shape, concentration, and distribution of nanoparticles so as to develop high-quality metal-based nanocomposites.

### Current Density

**Fig. 7:**
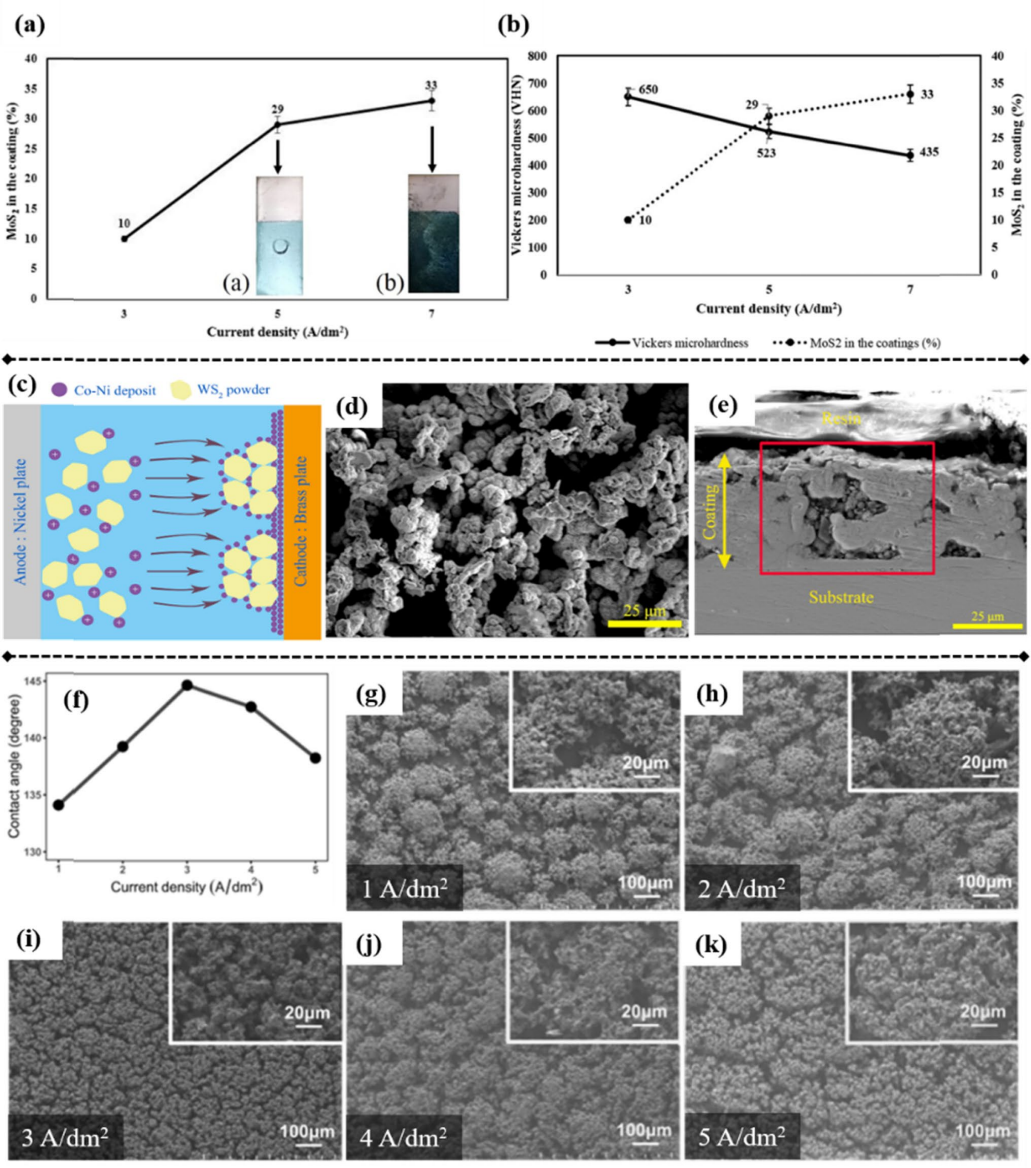
Variation in MoS_2_ content within the Ni matrix **a** and corresponding hardness **b** of Ni–MoS_2_ composite coatings under the influence of various current densities [127]. Codeposition mechanism of Co–Ni with WS_2_ powder **c**, which is proved by **d** surface morphology and **e** cross-section of Co–Ni–WS_2_ coating [93]. **f** Relationship between water contact angle (WCA) and applied current density for Ni–Co–WS_2_ composite coatings; **g**–**k** surface morphology variations observed at different current densities upon applying PC [143]

Applied current density plays a crucial role in influencing the internal stress, thickness, and particle incorporation content of nanocomposites. In general, appropriately increasing the applied current density would result in a higher particle content in the matrix. However, the performance of nanocomposites under the influence of high applied current density could be problematic. As shown in Fig. 7a, increasing current density to 3, 5, and 7 A/dm^2^ increased MoS_2_ content in the Ni matrix, rising from 10 to 33%, which was attributed to enhanced electric attraction and increased amount of particles controlled by charge transfer [127]. Accordingly, the COF decreased in this study with the compromise of microhardness (decreased from 650 to 435 VHN). However, the quality of such coating fabricated using 7A/dm^2^ was not satisfactory, exhibiting a dull surface with increased Sa, attributed to particle aggregation on the coating surface. Consequently, 5 A/dm^2^ was selected as the optimum current density to fabricate the Ni–MoS_2_ composite coating with good uniformity and smooth surface. Importantly, the high current density can result in cracks in the nanocomposites, as the internal stress generated during the codeposition exceeds the fracture strength of the nanocomposites [137, 138]. Therefore, current density should be optimized alongside temperature and pH to ensure low internal stress of the nanocomposites.

Current density and its distribution also affect the composites’ uniformity. Poor current distribution would result in irregularities on the metal composites, where metal ions tend to preferentially attach to specific locations on the cathode surface. Liu et al. observed that during the codeposition of Co–Ni and WS_2_ nanosheets, changes in the electric field caused by initially embedded WS_2_ altered the distribution of current density, resulting in higher current density around these WS_2_ sites (Fig. 7c) [93]. This increased current density attracted more Co and Ni ions, resulting in preferential deposition of Co–Ni around WS_2_ sites and forming Co–Ni/WS_2_ bulges on the composite coating’s surface (Fig. 7d). Cross-sectional analysis of these nanocomposite coatings further supported this observation (Fig. 7e). A similar phenomenon was reported by Zhou et al. [139], wherein high current density was observed surrounding nanoparticles, facilitating their selective growth.

The traditional DC electrodeposition applies a continuous, steady current, resulting in a constant metal ion deposition rate. In contrast, pulse current (PC) electrodeposition, which employs a periodic rectangular waveform, enables precise control of the electrocrystallization process, making it effective for fabricating nanocomposites with unique structures and properties [140]. PC applies current in pulses (on and off cycles), allowing for controlled deposition with rest periods that replenish ions at the electrode’s surface. This technique has been successfully used to fabricate nanocrystalline metals, alloys, and composites. Compared with DC plating, PC can produce finer grains and a denser microstructure, as pulsing can limit grain growth and facilitate more uniform nucleation, resulting in smoother nanocomposites with smaller grains. Therefore, PC electrodeposition offers notable advantages including producing ultrafine-grained structures and yielding deposits with a more homogeneous surface appearance. These improvements result in enhanced performance, including enhanced hardness [141], wear resistance [142], and hydrophobicity [143]. By adjusting key parameters, such as pulse frequency, pulse duration, peak current density, average current density, and duty cycle, PC electrodeposition allows for precise control of the microstructure and composition of nanocomposites, resulting in superior performance and customized material characteristics.

For instance, Wand et al. observed the PC-prepared Ni–Co–WS_2_ composite coatings showed more complex and finer grain structures with high and low undulations, whereas DC-based preparation resulted in flatter composite coatings [143]. In addition, they observed that upon increasing current density during PC, WCA first increased and then decreased (Fig. 7f). WCA achieved the highest value of 144.7° at 3 A/dm^2^. Such change in surface hydrophobicity was attributed to differences in surface morphology at various applied current densities. At 3 A/dm^2^, clusters on the coating surface were dense and evenly distributed, whereas they appeared loose or uneven at lower and higher current densities (Fig. 7 g–k). Moreover, Li et al. employed PC to prepare Ni–P–PTFE nanocomposite coatings with improved surface quality, enhanced micro hardness due to grain refinement, and notably reduced COF [144]. The key benefits of using PC for fabricating Ni-based nanocomposites can be summarized as grain refinement, improved particle dispersion, enhanced mechanical performance, improved surface smoothness, and reduced internal stress.

### Bath Agitation

The type and intensity of bath agitation affect composite quality by affecting particle dispersion/transportation and stability in the electrolyte. Such dispersion is often achieved by electrode movement [145], motor-driven blades [105], magnetic stirrers [127], or ultrasonic treatment via probe [14, 146]/bath [147].

**Fig. 8:**
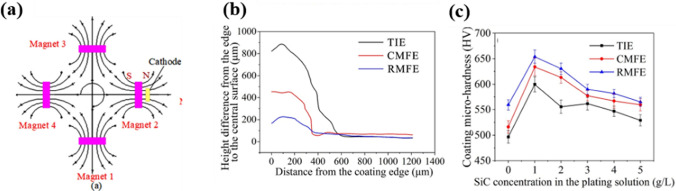
**a** Initial position of the magnet in a rotating magnetic field; **b** edge coating profiles for various electrodeposition methods, including traditional immersion electrodeposition, constant magnetic field-assisted immersion electrodeposition, and rotating magnetic field-assisted immersion electrodeposition; **c** microhardness values of Ni–SiC composite coatings across various electrodeposition techniques [145]

Chang et al. used a rotating disk electrode to codeposit MoS_2_ with Ni, and they observed that the incorporation content of MoS_2_ in the Ni–MoS_2_ composites increased by increasing the rotating speed from 500 to 1500 rpm [148]. Ji et al. applied a rotating magnetic field in the codeposition of SiC nanoparticles with Ni (Fig. 8a), which could attract charged particles within the plating solution. Consequently, the particles surrounding the electrode were evenly distributed, effectively addressing issues such as irregularities along the field’s edges and tip discharge (Fig. 8b) [145]. The dispersion effect of SiC was high under the influence of this rotating magnetic field, leading to a smaller grain size in Ni–SiC nanocomposites and thus resulting in higher microhardness (Fig. 8c).

**Fig. 9:**
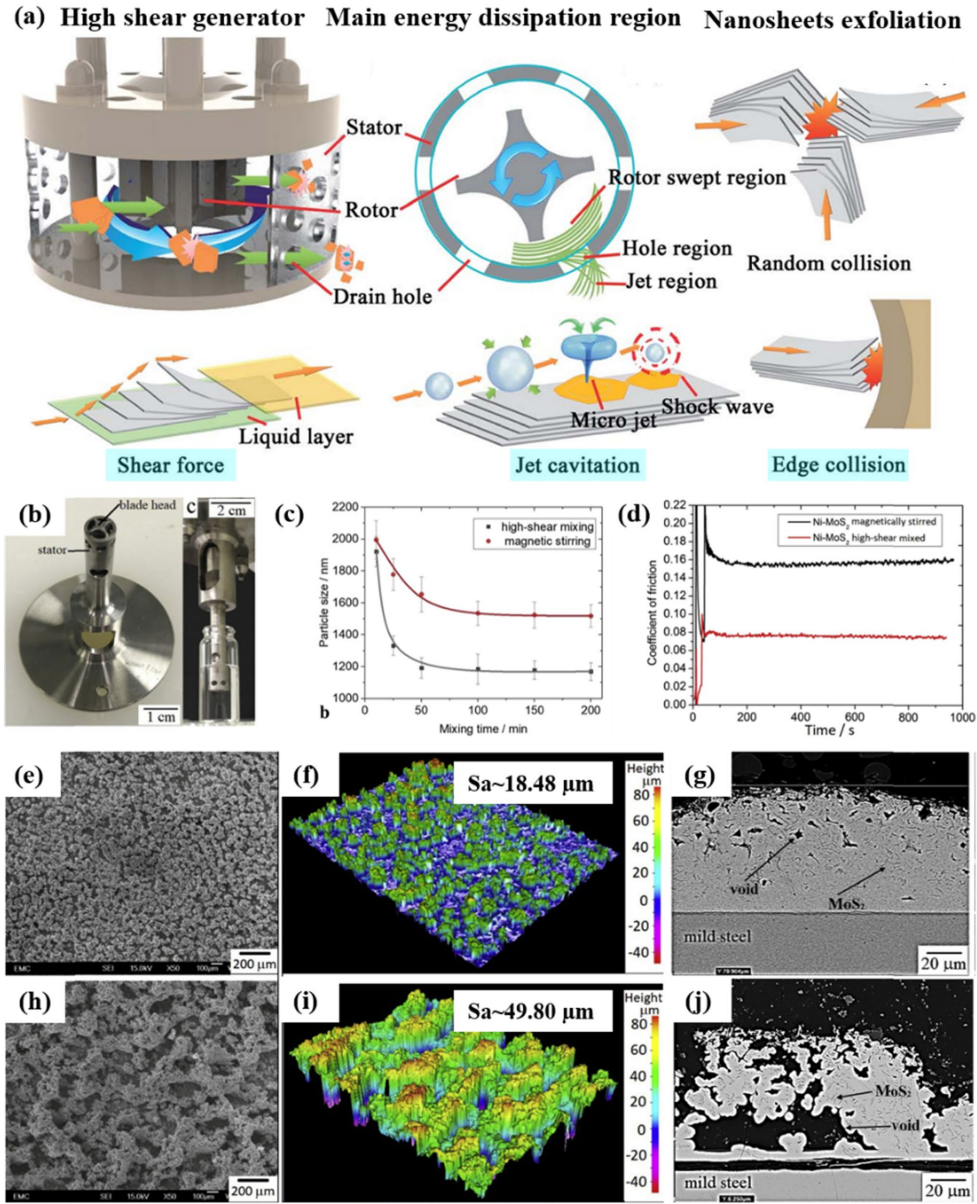
**a** High shear generator, with the sectional view of main energy dissipation regions, and three working mechanisms for nanosheet exfoliation, namely shear force, collision, and jet cavitation [149] **b** High shear mixer blade used for blending MoS_2_ nanoparticles prior to codeposition with Ni; **c** variation in MoS_2_ particle size with mixing time, comparing high shear mixing at 8000 rev/min with magnetic stirring at 2000 rev/min; **d** COF comparison between Ni–MoS_2_ composites fabricated via high shear mixing versus those fabricated via magnetic stirring; **e**–**g** surface morphology, profile, and cross-sectional morphology of Ni–MoS_2_ composites dispersed via high shear mixing; **h**–**j** corresponding features of Ni–MoS_2_ composites dispersed using magnetic stirring [105]

High shear mixing is a process that creates large fluid velocity differences around a fast-spinning rotor (speed: 10–50 m/s). This rotor is positioned close to a stator, with a narrow gap of 100–3000 µm between them, creating a zone of intense shear forces for fluids passing through (Fig. 9a) [149]. The high shear generator operates through three primary mechanisms: shear force, collision effects, and jet cavitation [150]. In nanoparticle dispersion, key operational parameters include mixing speed and mixing time. For dispersing nanoparticles such as graphene nanosheets, the intense and rapid fluid motion due to high shear mixing can create high physical interactions with these nanosheets, thus effectively overcoming the van der Waals forces between them [149].

Zhou et al. used high-shear mixing to disperse MoS_2_ nanosheets and avoid agglomerates in the bath (Fig. 9b) [105]. This technique demonstrated its effectiveness in reducing the particle size of MoS_2_ within the electrolyte solution (Fig. 9c), particularly compared with magnetic stirring, and resulted in a greatly reduced COF of the nanocomposite coating fabricated (Fig. 9d). In their study, the coating produced via high shear mixing had densely packed nodular structures with a low Sa of ~ 18.48 µm, while the one fabricated via magnetic stirring had porous structures with a rough surface (Sa of ~ 49.80 µm). Moreover, thanks to the high dispersion efficiency of high shear mixing, the morphology of the cross-section also exhibited a compact layer when using this method, while magnetic stirring generated extensive porosities, leading to a rough coating surface.

**Fig. 10:**
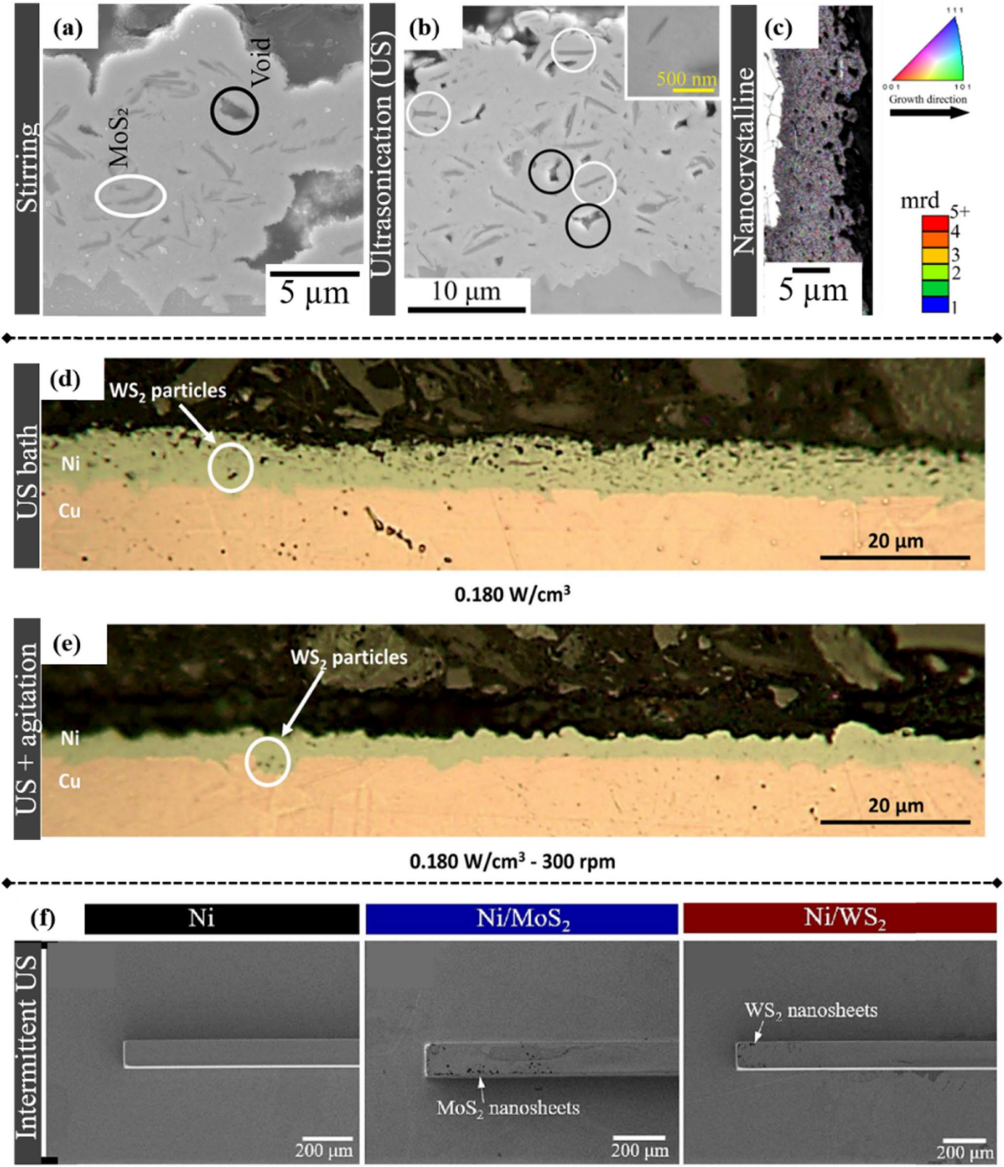
Ni–MoS_2_ composite coating dispersed via stirring **a** and ultrasonication **b**, and the orientation map of Ni–MoS_2_ coating treated with an ultrasonic horn [94] **c**. Cross-section of Ni–WS2 nanocomposite coatings fabricated via ultrasonic bath **d** and those fabricated via a combination of ultrasonication and mechanical agitation **e** [147]. Surface morphology of Ni molds compared with Ni–MoS_2_/WS_2_ nanocomposite molds synthesized via intermittent ultrasonication **f**

Pinate et al. used an ultrasonic horn to disperse MoS_2_ nanosheets, and the result demonstrated that the Ni–MoS_2_ coating obtained via stirring dispersion showed extensive porosities and irregular topography, while the coating obtained via ultrasonic dispersion had porosities with considerably smaller dimensions and decreased quantity (Figs. 10 a and b) [94]. With such ultrasonic agitation, a nanocrystalline microstructure was observed on the Ni–MoS_2_ nanocomposites, greatly improving microhardness and reducing COF (Fig. 10 c). A similar nanocrystalline formation was observed by Shourije et al. [127]. Tudela et al. used an ultrasonic bath combined with an overhead stirrer for dispersing WS_2_ nanosheets during electrodeposition. However, they observed that the combined use of ultrasonication and mechanical agitation resulted in thinner coating (Figs. 10 d and e), attributed to the particles glancing away from the cathode’s surface [147]. Guan et al. also employed ultrasonication during electrodeposition for fabricating 4-inch Ni–MoS_2_/WS_2_ composite molds [14]. Intermittent ultrasonication facilitates the long-term stability of MoS_2_/WS_2_ nanosheets in the electrolyte, as indicated by a polydispersity index (PDI) of less than 0.3 for both types of nanosheets. After codeposition, MoS_2_/WS_2_ nanosheets were observed on the surface of the composite molds (Fig. 10 f), affording lubrication properties to such composite molds.

While dispersion methods have shown promise in preventing aggregation of lubricating nanoparticles in electrolyte solutions, their widespread implementation beyond laboratory settings remains limited. In fact, achieving large-scale production while mitigating nanoparticle agglomeration in substantial volumes of electrolyte solution poses a notable hurdle. Innovative approaches are needed to address this challenge. One such avenue for exploration involves the use of surfactant mixtures. By combining different types of surfactants with complementary properties, such as varying molecular weights or surface activities, dispersion and stability of nanoparticles within the electrolyte may be enhanced on a larger scale. This approach capitalizes on the synergistic effects of multiple surfactants to achieve superior dispersion efficiency of nanosheets.

In addition, a combination of dispersion methods can offer a viable solution. Through the integration of various techniques, such as ultrasonication, mechanical stirring, or magnetic field-assisted dispersion, in a coordinated manner, it may be feasible to achieve more effective dispersion and prevent agglomeration across larger volumes of electrolyte solution. Furthermore, intermittent treatment methods can be explored to maintain nanoparticle dispersion over extended periods. This approach involves periodic agitation or redispersion of the solution to counteract particle settling or agglomeration, thereby preserving dispersion uniformity over time.

Other than the abovementioned three critical factors, electrolyte temperature [121, 151], pH [152], plating time [153, 154], surfactant type/concentration [29], and other variables also affect properties of the resulting composites, all of which have been recently discussed by Sreekumar et al. [82] and Raghavendra et al. [155].

## Properties of Lubricating Ni-Based Nanocomposites

### Microhardness

The enhancement of microhardness in metal nanocomposites can be attributed to three primary factors: particle strengthening, dispersion strengthening, and grain refinement. The presence of hard nanoparticles and their proportional increase in volume percentage would yield particle-strengthening effects on the metal nanocomposites. In addition, embedding ultrafine nanoparticles, as opposed to micro-sized particles, facilitates dispersion strengthening. Grain refinement is achieved through small grain nucleation within the nanocomposites, resulting in structural refinement. Consequently, this refinement contributes to an increase in hardness, impeding the movement of dislocations within the metal matrix. Typically, the microhardness of nanocomposites increases with particle content up to an optimal incorporation level, exceeding which the enhancement may become limited or diminished.

**Fig. 11:**
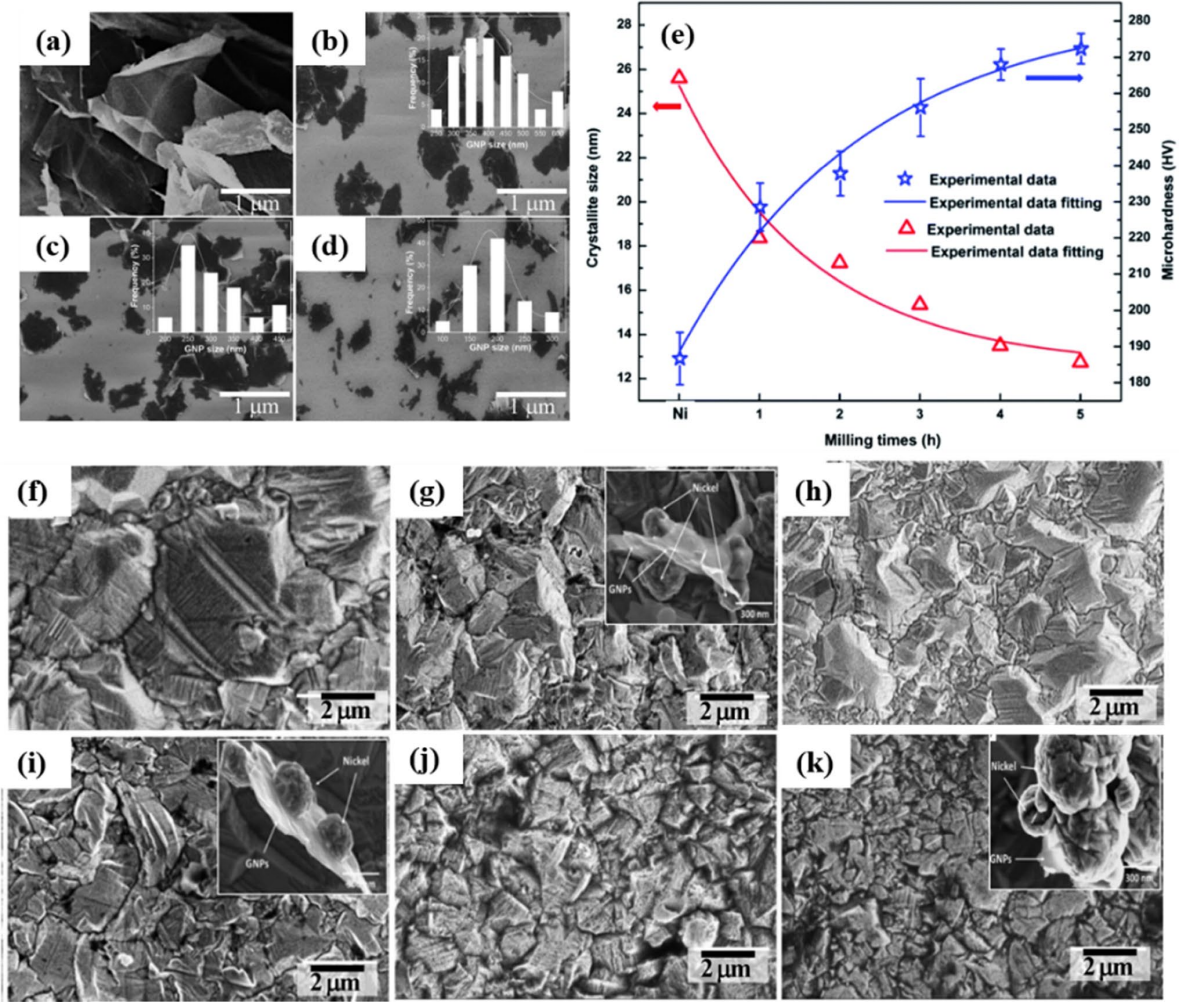
Graphene nanoplatelets (GNPs) before **a** and after ball milling for various durations: **b** 1 h, **c** 3 h, and **d** 5 h, showcasing distinct changes observed via SEM imaging. **e** Microhardness and crystallite size of Ni and Ni/GNP coatings, fabricated using GNPs milled for 1, 2, 3, 4, and 5 h, respectively. SEM images depict the **f** Ni coating and **g**–**k** various Ni/GNP nanocomposite coatings fabricated using GNPs milled for 1, 2, 3, 4, and 5 h, respectively [103]

Van Hau et al. highlighted the notable effect of dispersion strengthening on the microhardness of Ni–graphene nanocomposite coatings [103]. In their study, ball milling was employed to prepare GNPs of varying sizes via adjustment of milling time. GNP sizes effectively reduced with ball milling time increasing from 1 to 5 h (Fig. 11 a–d). Accordingly, the smaller dimensions of GNPs were critical to reducing the crystallite size of the resultant nanocomposites, notably enhancing their microhardness (Fig. 11e). The hardness enhancement mechanism was clearly revealed in this study: the introduction of smaller GNPs (~ 200 nm) into the Ni matrix notably reduced the grain sizes of the nanocomposites from several microns to less than one micron. SEM images revealed the different mechanisms of Ni crystalline formation in relation to various GNP sizes. For larger GNP sizes, Ni crystal nucleation formed and grew onto the GNP surfaces (Fig. 11 g–j). In contrast, GNPs with dimensions of 200 nm intercalated within the forming crystal nucleation (Fig. 11 k), preventing oriented crystal growth and reducing nickel grain size. The increased hardness was attributed to the uniform dispersion of small GNPs within the Ni matrix, as well as a decrease in Ni grain size through the use of milled GNPs. As per the Hall–Petch equation [103], a smaller grain size would result in higher microhardness, reiterating that small GNPs have a strengthening effect on nickel composites.

**Fig. 12:**
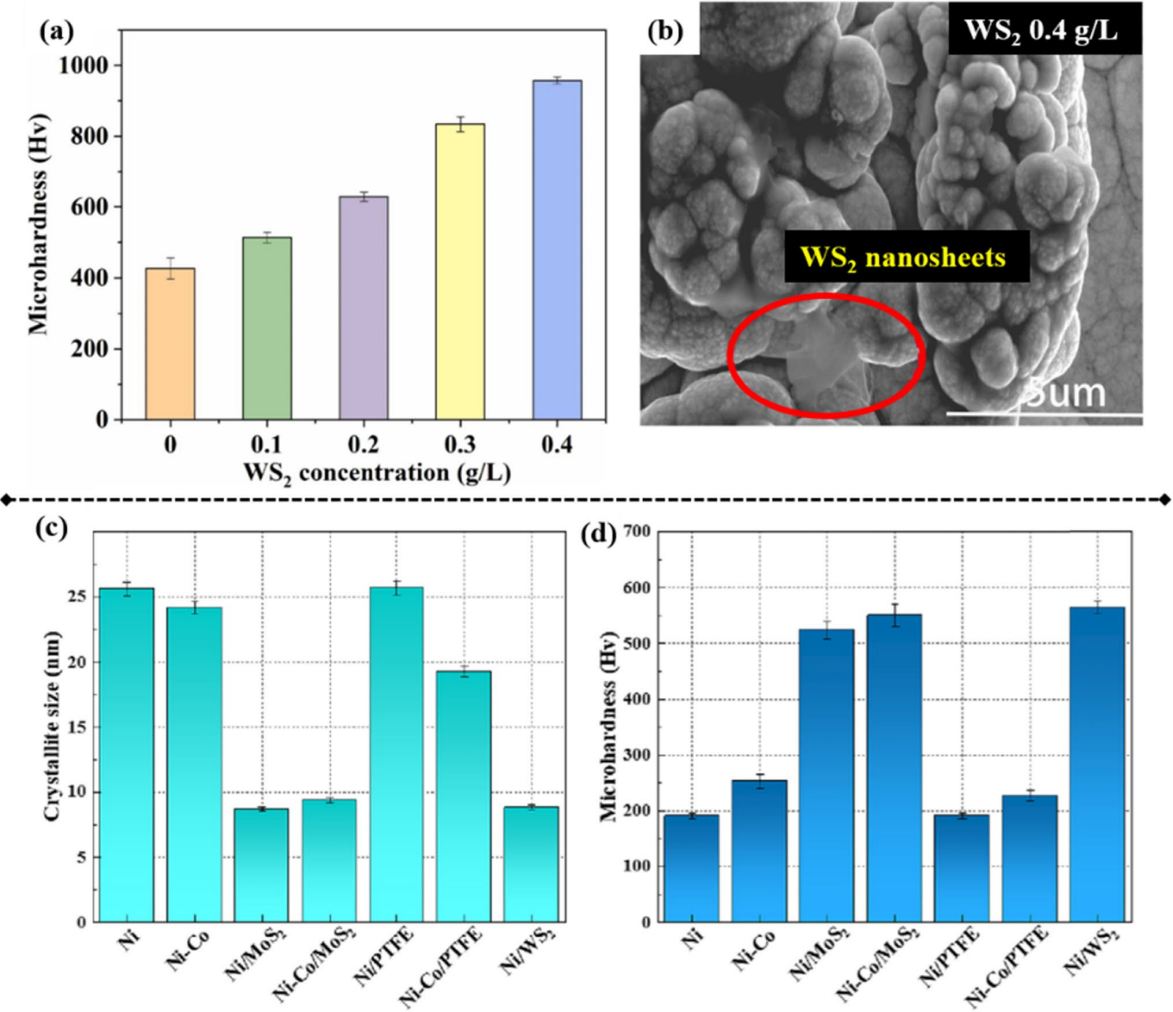
**a** Microhardness measurements of Ni–W/WS_2_ composite coatings at varying WS_2_ concentrations. **b** Surface morphology of a Ni–W/WS_2_ coating at 0.4 g/L WS_2_, showing WS_2_ nanosheets forming an accumulation layer [125]. **c**, **d** Crystallite size and microhardness of Ni-based nanocomposite molds incorporating various nanomaterials, with cobalt being the secondary phase [111]

Li et al. observed that incorporating Al_2_O_3_ nanoparticles at concentrations of less than 20 g/L notably enhanced the microhardness and wear resistance of Ni–B–Al_2_O_3_ nanocomposite coatings. This improvement was attributed to grain refinement and dispersion strengthening [133]. Similarly, Liu et al. investigated the effect of WS_2_ concentrations on the microhardness of Ni–W–WS_2_ composite coatings [125]. They observed that increasing WS_2_ concentrations from 0.1 to 0.4 g/L enhanced the microhardness from 514 to ~ 958 Hv (Fig. 12a). However, they also noted that at 0.4 g/L concentration, WS_2_ aggregates adhered to the coating surface, potentially compromising the coating’s performance (Fig. 12b). Consequently, 0.3 g/L was selected for fabrication of such nanocomposite coatings.

Guan et al. investigated the effect of various micro/nanoparticles on the microhardness of Ni–Co alloy composite molds [111]. Their findings revealed that incorporating micro-sized PTFE did not reduce the crystallite size of the resulting composites, resulting in lower microhardness than that of pure Ni mold (Fig. 12 c and d). The incorporation of second-phase cobalt reduced the crystallite size, thus enhancing the hardness. The addition of MoS_2_/WS_2_ nanoparticles further enhanced hardness via dispersion strengthening, grain refinement, and particle strengthening. Subsequently, nano-sized PTFE particles were introduced into the Ni matrix to fabricate the Ni–PTFE nanocomposite mold, and using such molds has resulted in greatly enhanced microhardness versus using microcomposites, attributed to particle strengthening and dispersion strengthening [114].

Other than particle type, concentration, size, and other variables also affect the microhardness of nanocomposites. Additives such as saccharin have been observed to effectively decrease the grain size of the Ni matrix, thereby enhancing its hardness [71, 156, 157]. Similarly, polyethene glycol with higher molecular weight decreased the grain size of Ni, thus enhancing its microhardness [158]. Surfactants could affect the surface charge/movement/dispersion effect/incorporation content of charged nanoparticles, hence affecting the microhardness of the resulting nanocomposites [29, 159].

PC may be used to enhance the microhardness of Ni nanocomposites, as studied by Chronopoulou et al.[120]. A more uniform Ni–graphene coating was developed by applying PC versus DC in their study. Similarly, PC methods have been suggested to extend relaxation periods, facilitating the adsorption of anions or other species onto the surface of composites [160]. This adsorption phenomenon initiates renucleation processes during subsequent on-pulses, resulting in grain refinement and enhanced structural defects. Consequently, this would result in higher microhardness values in metal nanocomposites.

### Surface Roughness and Wettability

Sa and surface chemistry are critical factors influencing the wettability of Ni nanocomposites. Wettability is often assessed by measurement of WCA on solid surfaces. Higher Sa can enhance mechanical interlocking during relative surface movement, thereby increasing COF and wear rate. Typically, nanocomposites exhibit lower Sa than microcomposites, resulting in reduced friction. Moreover, it has been observed that increasing the Sa of Ni-based nanocomposites creates “air pockets” that repel water from the surface, resulting in enhanced hydrophobicity [161, 162]. Surface structures such as re‑entrant structures have also attracted attention toward creating hydrophobic surfaces [163]. In summary, optimizing Sa and chemistry is critical to altering the wettability of Ni-based nanocomposites.

**Fig. 13:**
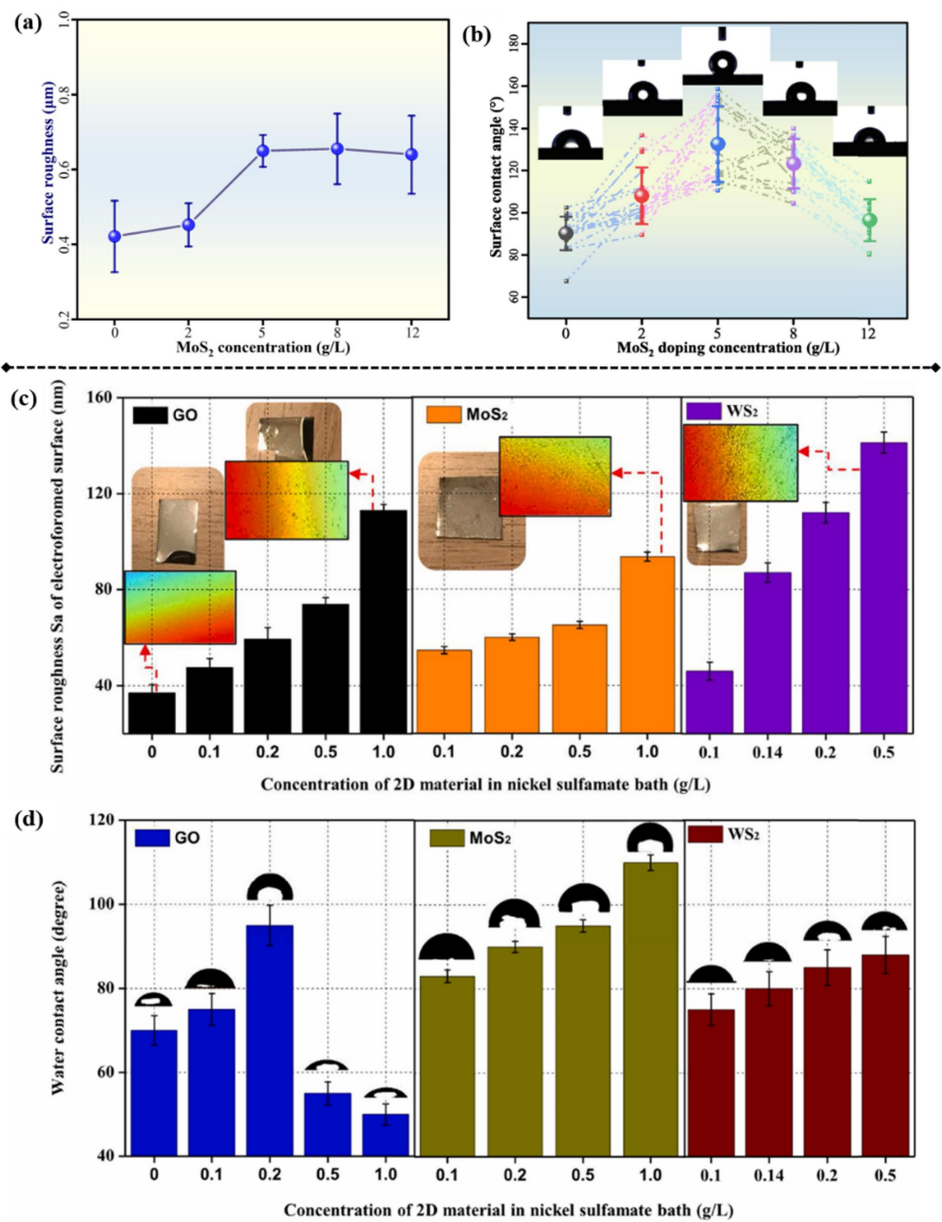
**a** Surface roughnesses (Sa) and **b** WCAs of Ni–WC–MoS_2_ nanocomposite coatings at varying MoS_2_ concentrations [164]. **c** Sa and **d** WCA of Ni–GO, Ni–MoS_2_, and Ni–WS_2_ composites incorporating nanoparticles at various concentrations [51]

Ren et al. investigated the effect of varying MoS_2_ concentrations on the surface properties of Ni–WC–MoS_2_ composite coatings [164]. As per their findings (Fig. 13a), as particle concentration in the bath was increased from 2 to 12 g/L, Sa of the coatings initially rose prior to stabilizing. The highest Sa value of 0.655 µm was observed at 8 g/L particle concentration. In addition, micro–nano structures such as “cauliflowers” and “nodules” were identified on the surface, contributing to the increase in Sa. Correspondingly, the WCA of the coatings increased and then decreased with the rise in MoS_2_ concentration (Fig. 13b). Notably, when particle concentration was too high, inter-nanoparticle aggregates were formed, and the surfactant–nanoparticle equilibrium was no longer present at such high concentrations. Therefore, a proper nanoparticle concentration should be selected for effective improvement of surface hydrophobicity. Similarly, MoS_2_ particles (~ 800 nm) and Ni particles (~ 100 nm) were electrodeposited with Ni–Co alloy by Jiang et al.[165]. The results demonstrated that coral-like micro/nanostructures led to a roughened coating surface and that the composite coating exhibited superhydrophobicity with remarkable self-cleaning properties.

Zhang et al. introduced various nanoparticles into the Ni matrix for the development of Ni-based nanocomposite molds [51]. Figure 13c shows the increase in Sa caused by increased particle concentration. Consequently, the WCA of Ni–WS_2_/MoS_2_ nanocomposite molds also increased because of the change in Sa and more hydrophobic particles embedded in the Ni matrix (Fig. 13d). For Ni–GO composites, when GO concentration was less than 0.2 g/L, the contact angle increased and peaked at ~ 95°, indicating a slightly hydrophobic surface (Fig. 13d). However, the contact angle decreased to approximately 50° as GO concentration was raised to 0.5 and 1.0 g/L, representing a more hydrophilic surface than that of pure Ni. Therefore, we can conclude that at GO concentrations less than 0.2 g/L, the surface microstructure primarily influenced the surface wettability of the composites. In contrast, at higher GO concentrations of 0.5 and 1.0 g/L, the surface wettability was predominantly governed by GO content, which was attributed to the hydrophilicity of GO.

Similarly, GO was reported to cause the surfaces of Ni–GO coatings hydrophilic after electrodeposition [42, 161]. This result demonstrated the importance of selecting proper nanomaterials for altering the surface wettability of the resulting nanocomposites. For example, when developing a self-lubricating mold tool, hydrophobic nanoparticles could provide lower surface energy for the resulting nanocomposites versus hydrophilic nanoparticles, which would be critical for successful demolding.

**Fig. 14:**
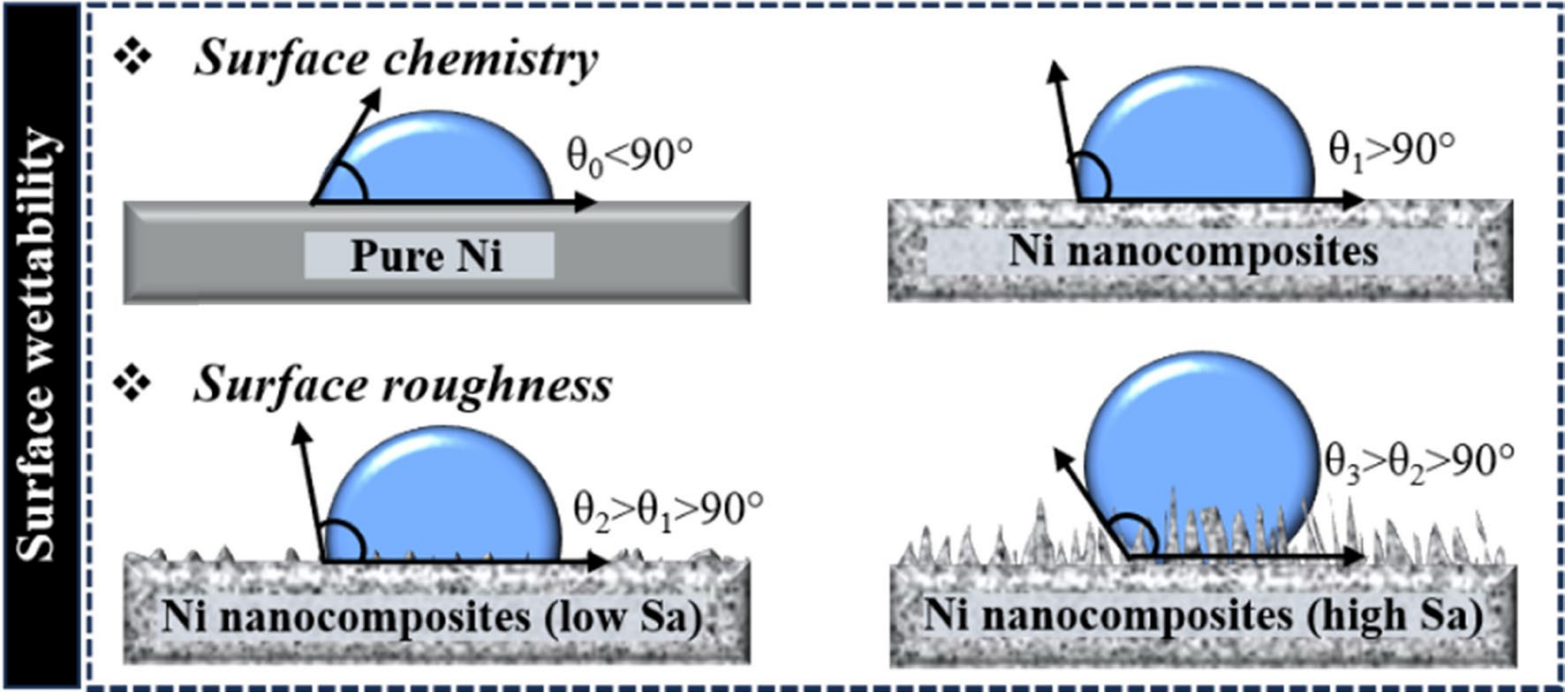
Mechanism of WCA change caused by surface chemistry modification or Sa increase

PTFE micro/nanoparticles were incorporated into the Ni matrix, and micro-sized particles were observed to roughen the surface of Ni composites [114], while Ni–PTFE nanocomposites showed low Sa. However, the WCA remained nearly the same for both the Ni–PTFE microcomposite (96.4°) and nanocomposite (97.1°), primarily because of the hydrophobic properties afforded by PTFE particles. The wettability change mechanism is shown in Fig. 14. Pure Ni has a WCA of ~ 78°–87°, as per different Sa values [82]. The dispersion effect is typically good when low concentrations of hydrophobic nanoparticles are introduced into the Ni matrix. The composite surface becomes hydrophobic (WCA > 90°) because of the hydrophobic nature of certain nanoparticles. In this situation, the Sa is low, and the WCA remains low but still higher than 90°. Once the particle concentration is increased or the dispersion effect is reduced, a higher Sa is achieved on nanocomposites and WCA will also potentially increase because of the air entrapped between the water and nanocomposite. Such surface chemistry and structures enable nanocomposites with self-cleaning [165, 166], anti-corrosion [166, 167], or anti-icing properties [82].

### Tribological Performance

In pursuit of reducing the high energy losses attributed to friction and wear, innovative strategies have been proposed to foster a future energy-efficient economy. Friction and wear contribute to nearly one-fourth of global energy losses [168, 169], necessitating urgent attention to alleviate their adverse effects [170]. Notable research has been undertaken to explore the wear performances of various Ni-based composites under diverse sliding conditions (dry/wet) [64, 70, 94]. Lubrication is critical to reducing friction and wear in industrial applications. The primary function of lubricants is to reduce friction and wear, lower contact pressure, and prevent galling and seizure [82].

Ni-based nanocomposite coatings engineered for lubrication are mainly employed in load-bearing components of mechanical systems, such as gears, turbines, ball bearings, and other traction devices, emphasizing their importance in extending equipment life and enhancing performance [171]. Compared with pure Ni deposits and Ni-based microcomposites, Ni-based nanocomposites offer notable advantages, including reduced Sa, increased mechanical hardness, enhanced wear resistance, and greater friction reduction. For instance, Narasimman et al. observed that Ni–SiC nanocomposites incorporating 50-nm SiC particles exhibited higher wear and scratch resistances, with lower wear loss, versus Ni–SiC microcomposites containing 1 µm SiC particles [172]. This improvement was primarily attributed to the higher surface finish and higher microhardness of Ni–SiC nanocomposites. In contrast, composites with higher Sa and lower hardness typically experience more considerable wear loss and reduced wear resistance. Similarly, Guan et al. reported that Ni–PTFE nanocomposites with 117-nm PTFE particles showed higher wear resistance and greater friction reduction than Ni–PTFE microcomposites with 1-µm PTFE particles [114]. This performance enhancement was attributed to the nanocomposites’ lower Sa, increased hardness, and more uniform particle distribution.

Graphene, WS_2_, MoS_2_, and PTFE nanoparticles are the most widely used filler materials for enhancing the tribological performances of Ni-based nanocomposites. Static and dynamic frictions are affected by a complex interaction of chemical/physical/ physicochemical/mechanical properties of the contacting materials, as well as external conditions [151, 154, 155]. These factors encompass a range of molecular interactions [156], surface group nature and reactivity [157], crystallinity [158], Sa [159], and even scale [160]. Recently published lubrication mechanisms of Ni-based nanocomposites are presented in Table 3.

**Table 3:** Codeposition methods and COFs in recent Ni-based nanocomposites

Composites	Current type	Current density (A/dm^2^)	COF	counterpart	Lubrication mechanism	References
Ni-graphene coating	DC	1	Reduced from 0.43 to 0.14	GCr15 ball	Hardness enhancement, Tribo-film development	[77]
Ni-W-MoS_2_ coating	PC	0.2	Reduced from 0.27 to 0.14	Stainless steel ball	Incorporation of low concentrations lubricating MoS_2_, compact structure, good substrate adhesion	[173]
Ni-MoS_2_ coating	DC	4	Reduced from 0.6 to ~ 0.08	Bearing steel cylinder roller	MoS_2_ sheared into wear track and compact structure facilitates the formation of tribo-film	[105]
Ni-WS_2_ mould	DC	5	Reduced from 0.65 to 0.18	Stainless steel ball	Hardness enhancement; interlayer shear sliding of WS_2_ nanosheets, developing into self-lubricating film	[51]
Ni-W-WS_2_ coating	PC	5	Reduced from 0.42 to 0.14	Si_3_N_4_ ball	Hardness enhancement, better load-carrying capacity and low shear strength of WS_2_, friction film development	[125]
Ni-PTFE mould	DC	5	Reduced from 0.46 to 0.22	PMMA pin	Hardness enhancement, low surface energy, low surface roughness	[114]
Ni-W-PTFE	PC	1.2	Reduced from 0.78 to ~ 0.2	Scratch tester	PTFE filled the pores in the coating, reducing the Sa, solid lubricant film development	[129]

Lubrication is critical to reducing friction and wear between contacting surfaces, whether they are stationary or relatively moving. To achieve effective lubrication and minimize wear on composites, several widely used methods have been proposed:

- “Tribofilm” formation. An effective method is to develop a “tribofilm” between these surfaces to divide the contacting surfaces [152], which can efficiently minimize friction and wear, thereby reducing energy losses and enhancing operational efficiency.
- Hardness enhancement. Another method is to enhance the microhardness of the composites [77], as the wear rate is inversely related to the microhardness of the materials [174].
- The lubricating nature of incorporated nanoparticles [47, 68, 127].
- The incorporation of layered WS_2_ nanosheets into Ni–W alloy coatings has been shown to enhance the load-carrying capacity, effectively reducing the wear rate of composites [125].
- Reduced Sa. Achieving low Sa is a key advantage of nanocomposite coatings, attributed to the integration of nano-sized particles. For instance, the Ni–W–PTFE composite coating exhibits a notably lower COF than Ni–W alone. This improvement is attributed to PTFE effectively filling micro holes and cracks in the Ni–W matrix, thereby reducing overall Sa [129].
- Minimization of surface energy. Reducing surface energy can notably diminish adhesive wear, particularly when sliding against a polymer pin. The Ni–PTFE nanocomposite mold exemplifies this benefit, as its low surface energy greatly mitigates adhesive wear [114, 175].

PTFE nanoparticles were incorporated into a Ni–W alloy via pulse electrodeposition, resulting in composite coatings with chemically inert and naturally hydrophobic surfaces [129]. Increasing PTFE concentration greatly reduced the COF, with a slight enhancement in hardness detected, attributed to the soft and lubricating instinct of PTFE nanoparticles. Increasing the dispersibility of nano-sized PTFE in the electrolyte resulted in enhanced dispersion and strengthening effects, in turn increasing the microhardness of Ni–PTFE composites. The resulting high hardness and low surface energy of these Ni–PTFE nanocomposite molds provided remarkable wear resistance against polymer materials [114].

To achieve better nanoparticle dispersion in the electrolyte, Liu et al. used a polyvinylpyrrolidone-assisted reduction method to prepare Ni-reduced graphene oxide (RGO) deposits [176]. Incorporating a very low content of RGO nanosheets into the Ni matrix resulted in composites with low COF and enhanced anti-wear performance. This enhancement in tribological properties can be attributed to the grain refinement strengthening effect and formation of a protective nickel oxide film. In addition, graphene oxide was incorporated into the Ni matrix via ultrasonic-assisted electrodeposition to enhance hardness, reduce friction, and achieve self-lubrication [177]. Their study observed that GO concentration played a more notable role than ultrasonic irradiation in lowering the COF (from 0.45 to 0.3) and enhancing the wear resistance of the composite coatings.

Particle concentration was observed to notably affect the friction and wear properties of resulting composites. He et al. used different concentrations of MoS_2_ particles in the bath to develop Ni–P–MoS_2_ composite coatings [137]. As per their study, 10 g/L MoS_2_ resulted in the lowest COF of ~ 0.05, with 7.9 wt.% MoS_2_ detected in the composite coating. Further increasing particle content to 20 g/L lowered incorporation content and increased COF. Correspondingly, the Ni–P–MoS_2_ composites exhibited the highest wear resistance at 10 g/L MoS_2_ because of tribofilm formation and suppressed oxidation.

**Fig. 15:**
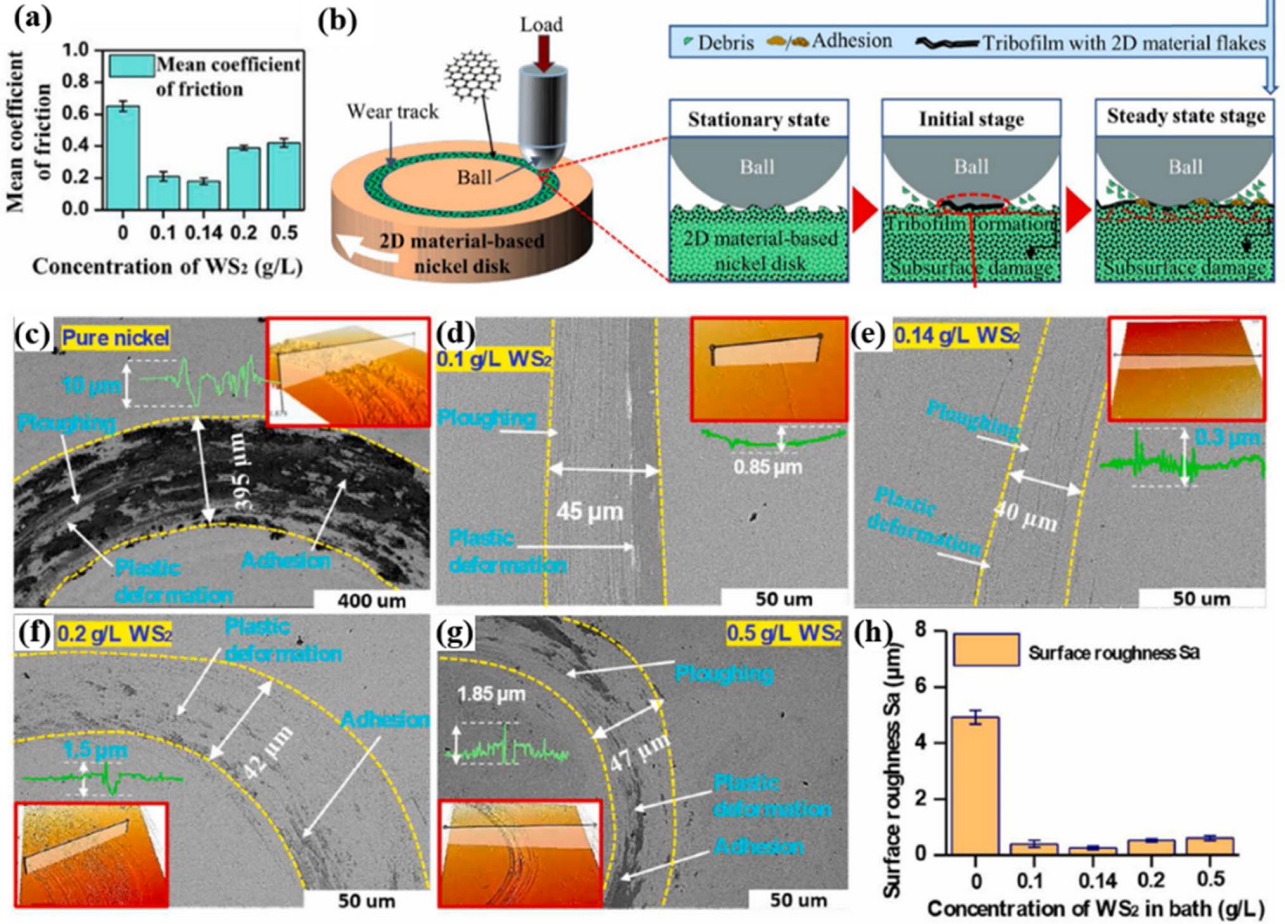
**a** COF of Ni–WS_2_ nanocomposites against a stainless-steel ball, measured under 1 N load and 31.8 rpm rotation. **b** Lubrication mechanism of Ni–WS_2_ nanocomposites when sliding against a stainless-steel ball. **c**–**g** Wear morphology of such nanocomposites with different particle concentrations. **h** Sa of worn surfaces at different WS_2_ concentrations [51]

Likewise, our previous study observed that COF was lowest when sliding against a stainless steel ball when 0.14 g/L WS_2_ nanoparticles were included in the Ni nanocomposite mold (Fig. 15a) [51]. Correspondingly, minimum wear was generated at this low particle concentration, along with the narrowest wear track and the most minor plastic deformation-induced wear (Fig. 15 c–g). Further increasing particle concentration resulted in severe aggregation of the lubricating nanoparticles. This agglomeration roughened the surface of the composite mold, hindering the formation of a lubrication film during contact with its counterpart (Fig. 15b).

Compared with MoS_2_, WS_2_ exhibits higher thermal stability even after being incorporated into the Ni matrix [82]. COFs of the Ni–WS_2_ composites remain stable, ranging between 0.01 and 0.03, even as temperature rises to 300 °C [69]. However, COF noticeably increases to 0.12 at 500 °C. This phenomenon is attributed to the gradual oxidation of WS_2_ into WO_3_, resulting in the loss of its lubricating properties. WO_3_ or MoO_3_ can often be observed on worn surfaces of metal composites, most commonly in the form of severe wear caused by metal counterparts such as stainless steel balls in ball-on-disk tests [178]. MoS_2_ can transform into MoO_3_ at ~ 102 °C in the presence of oxygen, which has been detected through Raman spectroscopy by Windom et al. [179]. Meanwhile, the effect of humidity was also examined in this study, demonstrating minimal influence on the oxidation process in comparison with exposure to dry air or oxygen [179].

In accordance with industrial application requirements, the counterpart material intended to interact with the composite should be carefully designed to thoroughly investigate the tribological performances of these nanocomposites. Our group developed Ni–MoS_2_/WS_2_ nanocomposite molds for defect-free fabrication of polymeric parts via micro hot embossing. Polymer pins (specifically PMMA and cyclo-olefin copolymer (COC) 8007) were used in the friction test to reveal the lubrication mechanism of such nanocomposite molds. This approach was selected because the Ni-based nanocomposite molds were intended to be used in contact with molded polymers during micro/nanofabrication, necessitating the study of the interaction between the molds and polymer materials. Thus, instead of using the conventional stainless ball, polymer pins were used to facilitate the investigation of the friction and wear resistance of the Ni-based nanocomposite molds. Comprehending the tribological performance (including friction and wear) of these nanocomposite molds against polymers is key to explaining their demolding behavior in micro/nanofabrication processes.

**Fig. 16:**
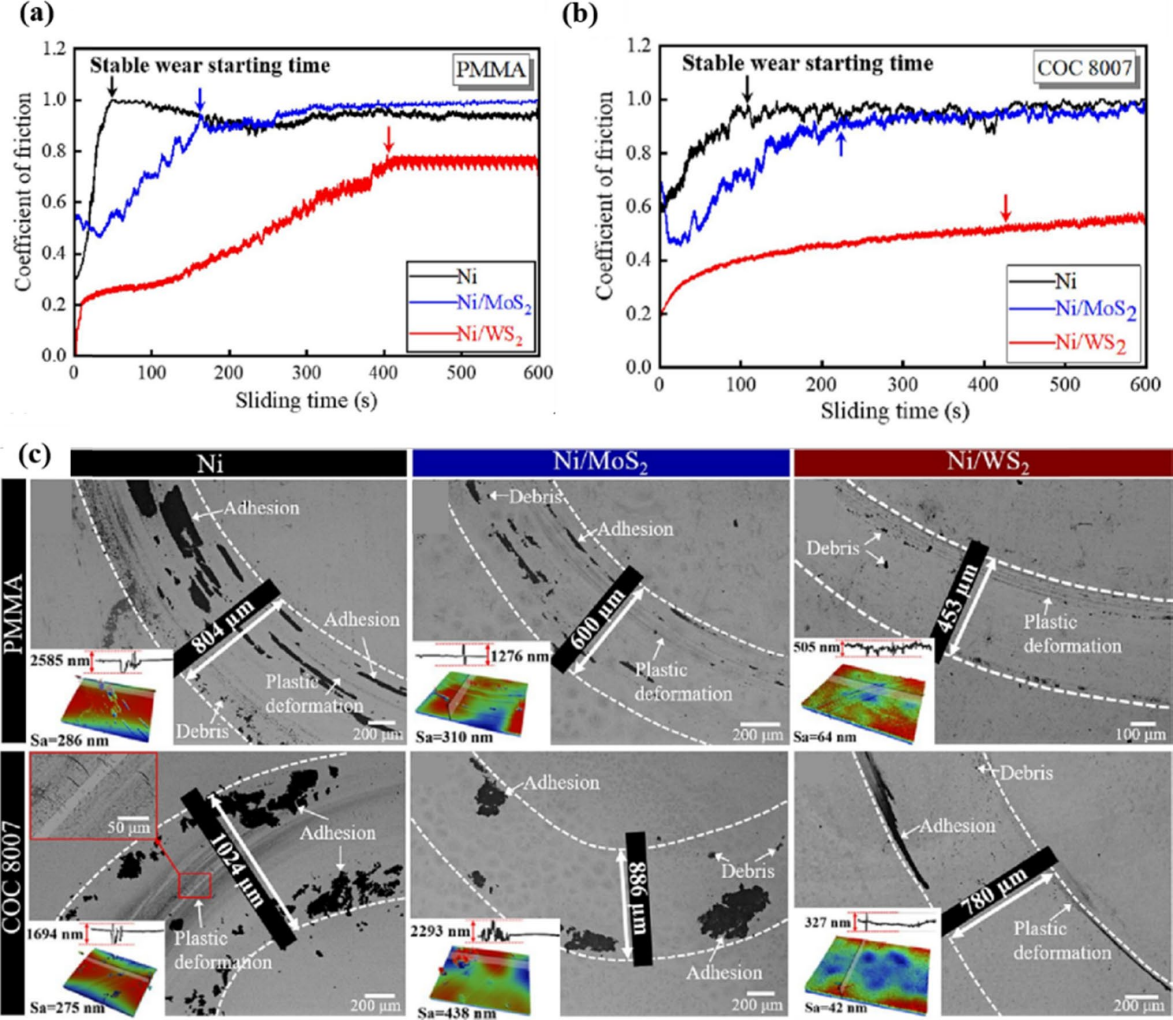
Frictional behavior of Ni–MoS_2_/WS_2_ nanocomposite molds against polymer pins: polymethylmethacrylate (PMMA) pin **a** and cyclo-olefin copolymer (COC) 8007 pin **b**, under 1 N load and 31.8 rpm rotation. **c** Wear morphology and profile of composite molds against polymer pins [14]

As shown in Fig. 16, the Ni–WS_2_ nanocomposite mold showed the lowest COF and highest wear resistance against polymer pins. In contrast to the “tribofilm” effect, the lubrication mechanism observed in this study was attributed to the low surface energy and enhanced hardness of these molds resulting from the inclusion of hard WS_2_ nanoparticles. Incorporation of MoS_2_/WS_2_ nanoparticles into the Ni mold matrix notably reduced adhesion-induced wear (Fig. 16c). Polymer pins have been used to examine the friction and wear characteristics of lubricating nanocomposites, which has also been reported in other studies [29, 111, 114, 175].

## Applications

**Fig. 17:**
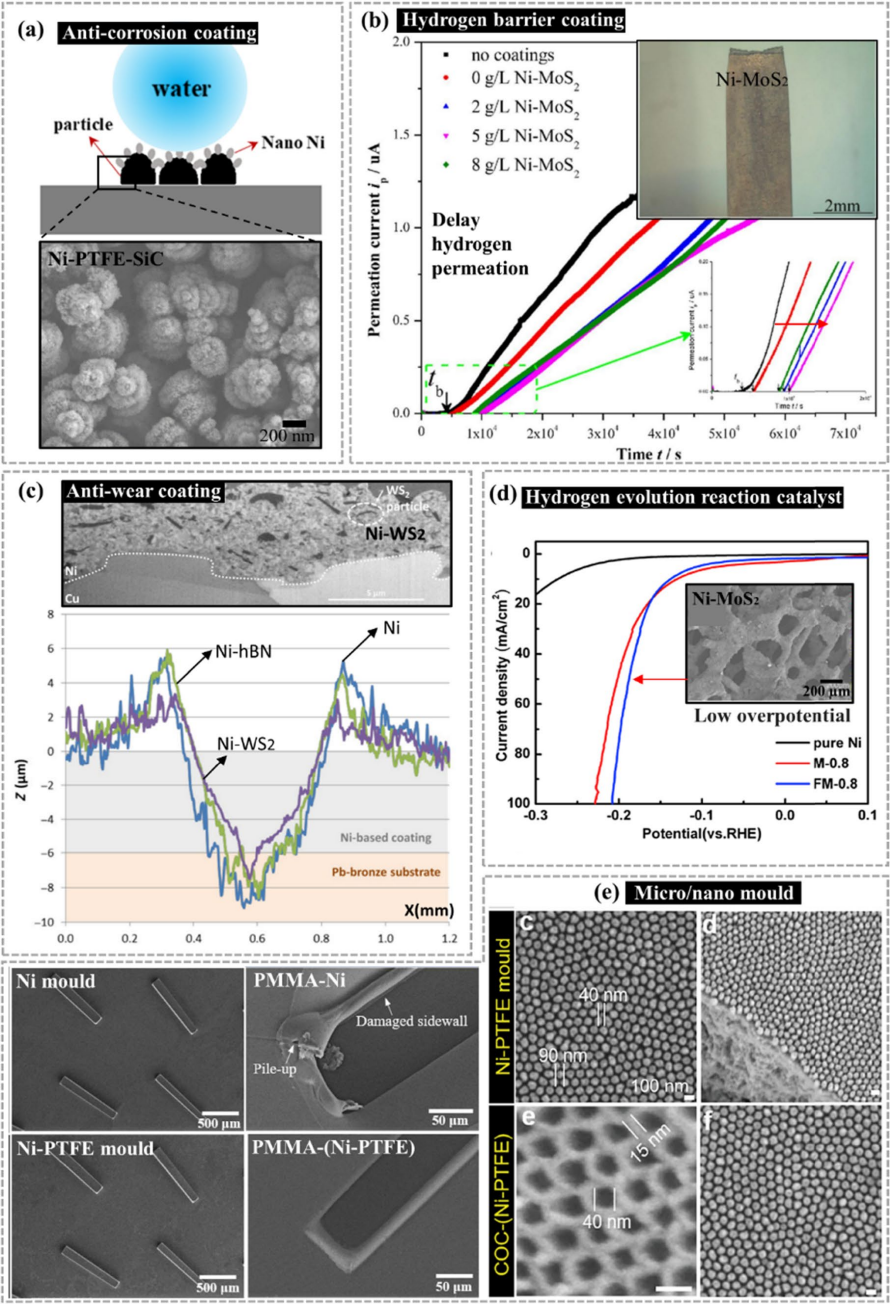
Applications of Ni-based nanocomposites: **a** Ni–PTFE–SiC nanocomposites as anti-corrosion coating [190]; **b** Ni–MoS_2_ nanocomposite coating against hydrogen embrittlement [181]; **c** Ni–WS_2_ nanocomposites as anti-wear coating [191]; **d** Ni–MoS_2_ nanocomposites as catalysts for the hydrogen evolution reaction [185]; **e** Micro/nano featured Ni–PTFE molds developed by our group: Ni–PTFE micro featured mold for hot embossing of PMMA chips [114] and Ni–PTFE nano structured mold for thermal nanoimprinting of COC film with features down to 15 nm [189]

Ni-based nanocomposites serve specific purposes, with the predominant application of enhancing wear resistance and tribological properties, particularly in the aerospace and automative sectors [1]. Their applications can vary based on properties desired and environmental conditions:

- Corrosion-resistant coatings: Nanocomposite coatings exhibit superhydrophobic properties because of the incorporation of WS_2_/PTFE nanoparticles (Fig. 17a) [143, 180]. They effectively reduce corrosion by minimizing the contact area between the metal nanocomposite substrate and corrosive liquids. Ni–graphene composites also exhibit anti-corrosion properties because of reduced exposure of the metallic surface area to corrosive environments [134]. Applications include marine environments and other industrial settings where exposure to aggressive chemicals can result in corrosion.
- Hydrogen barrier coatings: Ni–MoS_2_ nanocomposite coatings are used to protect quenching and partitioning steels from hydrogen embrittlement (Fig. 17b), largely reducing their hydrogen diffusion coefficient [181]. This improvement is attributed to high impermeability, refined grains, and reduced hydrogen diffusion of nanocomposite coatings. Incorporation of MoS₂ nanosheets into the Ni matrix results in refined grain structure, increasing the number of grain boundaries in the coatings and their impermeability. This barrier limits penetration by hydrogen atoms, preventing them from diffusing into the underlying steel.
- Wear-resistant coatings: Incorporating nanoparticles into Ni–P or Ni–Co alloy coatings notably enhances their hardness and wear resistance versus pure metals or conventional microcomposite coatings [102, 137, 143, 182, 183]. They are widely employed in engineering applications where durability and protection against wear and corrosion are essential, including ball valves, high-pressure compressor spacers, and engine bearings (Fig. 17c) [184].
- Electrocatalysts: Ni–MoS_2_ composite coatings have been effectively used as electrocatalysts for the hydrogen evolution reaction (Fig. 17d), exhibiting low overpotential and stable cycling performance [185]. Similarly, the electrocatalytic activity of Ni–Mo–WS_2_ composite electrodes is notable, attributed to surface area increase due to the incorporation of WS_2_ nanoparticles and the formation of an outer layer rich in Mo oxides [186].
- Low-friction coatings: Owing to PTFE’s low friction properties, it is integrated with Ni to form Ni–PTFE micro/nanocomposites. Applications include films/coatings on bearings, gears, and sliding components for which reduced friction and wear are critical [99, 187, 188].
- Micro featured composite mold tool: Our group has made notable progress in enhancing the performances of micro structured Ni mold tools by incorporating lubricating nanoparticles such as GO, WS_2_, MoS_2_, and PTFE into the Ni matrix via one-pot electroforming [14, 29, 51, 111, 114]. These microstructured Ni nanocomposite molds have shown lower friction and higher wear resistance against polymer materials, thereby reducing demolding forces and extending the lifespan of mold tools used in microfabrication of polymeric components via micro injection molding and hot embossing (Fig. 17e).
- Nano featured composite mold tool: Particularly noteworthy is our development of a permanent antisticking Ni–PTFE nano structured mold to address the demolding issues in nanoimprinting (Fig. 17e). By incorporating low-surface-tension PTFE nanoparticles, this Ni-PTFE mold is capable of imprinting densely packed nanostructures down to 100 nm for at least 20 cycles [189]. Notably, the Ni–PTFE mold maintained its structural integrity and surface cleanliness after repetitive nanoimprinting cycles. This reusable, high-resolution nanocomposite mold eliminated the need for anti-sticking treatments and ensured defect-free nanoimprinting, marking a remarkable achievement in the application of nanocomposites.

Development of Ni–PTFE nanocomposite molds holds notable promise for widespread industrial adoption, particularly in fields requiring high precision and durability, such as microfluidic chip production and nanoimprint lithography. The unique properties of these molds, including their extended tool life and reduced need for anti-sticking coatings, position them as a transformative innovation for precision manufacturing. The enhanced durability and antiadhesive properties of Ni-PTFE molds enable the fabrication of high-fidelity, defect-free components, which are essential in applications such as medical diagnostics, lab-on-a-chip devices, and biosensors. Moreover, the high wear resistance of Ni–PTFE molds results in fewer tool replacements and lower maintenance costs, directly translating into enhanced operational efficiency and cost-effectiveness. This is particularly impactful in industries where production downtime can result in notable financial losses.

## Conclusions and Future Perspectives

In summary, notable advancements have been made in the electrodeposition of Ni-based nanocomposites incorporating nanoparticles so as to achieve enhanced hardness, reduced friction, and improved wear and corrosion resistance. Integration of nano-sized particles has shown particular promise in refining the grain size and enhancing the overall mechanical properties in comparison with micro-sized particles. The development of effective codeposition techniques, including surfactant-assisted, ultrasonic, and high-shear methods, has been instrumental in addressing nanoparticle aggregation, resulting in more homogeneous particle distribution within the Ni matrix. However, the following limitations and challenges continue to persist:

- Transport mechanisms of nano-sized particles during the deposition process are still not fully understood, necessitating further research into their interactions with the matrix.
- The stability of nanoparticle dispersions over extended periods, particularly in large-scale production environments, remains a critical issue.
- Long-term effects of internal stress induced by high particle concentrations or large-sized particles during scaling up have been underexplored and may hinder industrial application.

Future research should focus on several key areas to advance the development and application of Ni-based nanocomposites:

- *Particle/matrix selection* Proper selection and introduction of nanoparticles of different types, shapes, and sizes into the Ni matrix may benefit the engineering performance of resulting nanocomposites. One can consider reinforcing Ni-based alloys with nanoparticles so as to achieve enhanced hardness and lubrication.
- *Dispersion* Prior to codeposition, ensuring nanoparticles are uniformly dispersed in the electrolyte solution is crucial. This step ensures that the embedded particles are evenly distributed throughout the deposits, which is often underappreciated in the existing literature. Studies regarding nanoparticle analysis (surface charge/particle size/particle distribution) within the electrolyte should be performed to fundamentally solve the problems associated with particle agglomeration.
- *Stability of dispersion* Stability of the nanoparticle dispersion in the electrolyte solution should be evaluated, including the manner in which particle size and distribution would change with sedimentation times. Typically, codeposition involves at least mechanical stirring/air agitation, which should be practically considered in sedimentation tests.
- *Reinforcement mechanism* High concentrations of particles (micro-, submicro-, or nano-sized) have been widely added to the bath for composite enhancement. However, the incorporation of well-dispersed, low-concentration, fine-sized nanoparticles can also enhance hardness/lubrication/wear resistance, which is often ignored in the literature.
- *Internal stress* Internal stress within composites is critical, particularly for scaling up composite coating/mold tools. Particles at high concentrations or those with large sizes would typically induce higher internal stress in the deposits. However, this characteristic has often been underestimated, making it challenging to extend laboratory results to industrial applications.
- *Evaluation* Based on conventional evaluation methods, more practical conditions should be applied during the test. For corrosion/friction/wear tests, practical conditions involving moderate/high temperatures can be applied, corresponding to harsh service environments such as those associated with bearings, seals, furnace components, etc. Particularly in the tribological test, the counterparts can be customized as per industrial applications, such as the use of polymer pins to evaluate a nanocomposite mold’s lubrication. Test conditions (dry/wet/in chemical solutions) should also be designed to suit the application of nanocomposites.
- *Sustainability* Maintenance and recycling/disposal of used surfactants/baths need to be considered in the plating process, as plating baths are supposed to serve for many years. Additive-free plating is favorable in terms of environmental concerns regarding the use of surfactants. However, the determination of residual nanoparticles/surfactants may resolve this issue, which involves replenishment of nanoparticles/surfactants in the bath.
- *Future applications* The most common future applications of Ni–WS₂/Ni–MoS₂ and Ni–PTFE nanocomposites include the following: 1) corrosion-resistant coatings, 2) protection of metal surfaces in harsh environments (such as environments associated with marine or chemical processing industries), 3) wear-resistant coatings (the high wear resistance of Ni–WS₂/MoS₂ nanocomposites makes them ideal for applications in cutting tools, machinery parts, and automotive components) 4) catalysts for the hydrogen evolution reaction, and 5) micro/nanocomposite mold tools with high performance and lubrication properties to make large-scale, defect-free replication of micro/nano surface features feasible.

## Data Availability

The authors declare that all data supporting the findings of this study are available within the article.
